# *Bacillus velezensis* M03 enhances peanut stress resistance and optimizes soil microecology, thereby increasing its resistance to *Ralstonia solanacearum*

**DOI:** 10.3389/fpls.2026.1788450

**Published:** 2026-04-10

**Authors:** Zongsheng Yuan, Sifan Wang, Xun Dong, Xiaoling Wang, Fang Liu

**Affiliations:** 1 Fujian Key Laboratory on Conservation and Sustainable Utilization of Marine Biodiversity, College of Geography and Oceanography, Minjiang University, Fuzhou, China; 2 State Key Laboratory of Ecological Pest Control for Fujian and Taiwan Crops, Fujian Agriculture and Forestry University, Fuzhou, China; 3 College of Life Sciences, Fujian Agriculture and Forestry University, Fuzhou, China; 4 College of Resources and Environment, Fujian Agriculture and Forestry University, Fuzhou, China

**Keywords:** *Bacillus velezensis*, peanut, *Ralstonia solanacearum*, soil microecology, stress resistance

## Abstract

**Introduction:**

Against the background of excessive application of chemical pesticides, which has caused the imbalance of soil microecosystems and the aggravation of pathogen resistance, the control of soil-borne diseases such as peanut bacterial wilt has become a major challenge in agricultural production. The traditional management mode relying on chemical pesticides is not only unsustainable, but also increasingly reveals its limitations.

**Methods:**

This study explores the effects of *Bacillus velezensis* M03 on enhancing peanut stress resistance and optimizing soil microecology, systematically evaluating its ability to improve peanut resistance to *Ralstonia solanacearum*.

**Results:**

It showed that *B. velezensis* M03 significantly inhibited the occurrence and spread of *R. solanacearum*, reducing the incidence of bacterial wilt from 50.00% to 16.67% three days after inoculation, with a disease index inhibition rate of 74.99%. *B. velezensis* M03 significantly increased the activities of SOD, POD, and CAT enzymes in peanut leaves, reduced MDA content, enhanced reactive oxygen species scavenging capacity, and mitigated membrane lipid peroxidation damage. *B. velezensis* M03 promoted soil nutrient transformation, significantly increasing the contents of nitrate nitrogen, available phosphorus, available potassium, and available sulfur, enhancing the activities of key soil enzymes such as phosphatase and urease, and optimizing nutrient availability. Microbial community analysis showed that *B. velezensis* M03 effectively inhibited the proliferation of *R. solanacearum*, promoted the enrichment of beneficial bacteria such as Streptomyces and Trichoderma, restored the bacterial network structure and modularity, and enhanced the functional stability of the microbial community.

**Discussion:**

*B. velezensis* M03 significantly enhanced the systemic resistance of peanuts to bacterial wilt by synergistically regulating plant physiological resistance, soil nutrient cycling, and microbial community structure, providing a theoretical basis and technical support for the microbial control of soil-borne diseases.

## Introduction

Peanuts (*Arachis hypogaea* L.) are an important oilseed and cash crop, playing a central role in ensuring the supply of edible oil and increasing farmers’ income in tropical and subtropical regions (Wang et al., 2024). Bacterial wilt, caused by *Ralstonia solanacearum*, has become one of the most serious soil-borne diseases threatening peanut cultivation (Zhao et al., 2025). The pathogen spreads through the soil and through the peanut’s vascular system, causing acute wilting and even death of the plant, resulting in large-scale yield reduction or crop failure (Chen et al., 2020). Currently, chemical control remains the main emergency measure. However, long-term and excessive application has induced multiple negative effects (Li et al., 2025). The pathogen’s resistance to pesticides is evolving rapidly (Cao et al., 2025), and pesticide residues cause soil and water pollution, disrupt the microecological balance, and threaten the safety of the soil ecological environment (Chen et al., 2020).

Biological control, particularly antagonists based on beneficial plant rhizosphere microbial resources, is considered the most promising breakthrough for replacing chemical pesticides due to its advantages such as strong targeting, good environmental compatibility, and low likelihood of inducing resistance (Bakr et al., 2025). The rhizosphere, as the core of plant-soil-microbe interactions, contains a highly diverse and functionally specific microbial pool (Raaijmakers et al., 2002). Among them, antagonistic *Pseudomonas*, *Bacillus*, and *Streptomyces* can significantly inhibit *R. solanacearum* through multiple mechanisms, including niche competition, antibiotic and siderophore secretion, induction of systemic resistance, and promotion of plant growth (Ling et al., 2020). However, existing research is mostly limited to verifying the antibacterial activity of strains. The multi-level, non-linear interactions between plant stress resistance and soil microbial communities, and their impact on rhizosphere microecology and function (Glandorf et al., 2001), still lack systematic analysis.

Rhizosphere microbial communities are not random aggregates, but complex ecological networks driven by co-evolution and exhibiting spatiotemporal heterogeneity. Their composition and dynamics directly influence soil nutrient cycling efficiency (Liu et al., 2023), disease suppression potential, and plant health (Klasek et al., 2022). The introduction of exogenous antagonistic bacteria may reshape community structure (species composition, abundance of key taxa, etc.) (Hu et al., 2016; Wei et al., 2015), functional potential (enzyme activity, expression of resistance genes) (Jousset et al., 2011), and interspecific interaction networks (co-occurrence, competition, synergy) through resource competition and cross-metabolism (Faust and Raes, 2012). Ignoring these potential non-targeting effects may induce microecological imbalances, weaken disease suppression effects, and even pose long-term hidden risks to soil health (Johansen et al., 2005).

Therefore, this study systematically evaluated the effects of *Bacillus velezensis* M03 on enhancing peanut stress resistance and optimizing soil microecology. It revealed the processes by which it enhances peanut stress resistance and optimizes soil microecology, providing a theoretical basis and practical support for developing probiotic-based green pest control technologies.

## Materials and methods

### Test materials

Peanut variety tested: Minhua 18 (moderately sensitive to bacterial wilt).
Cultivation substrate: A 1:1 mixture of peat moss (PINDSTRUP) and farmland soil.
Basic properties of the cultivation substrate: pH: 5.7; total nitrogen: 1.02 g/kg, total phosphorus: 0.25 g/kg, total potassium: 1.07 g/kg. No bacterial wilt was detected in the substrate.
Antagonistic bacteria: *B. velezensis* M03(accession number: CGMCC No.36560), an endophytic bacterium isolated and screened from the mangrove plant *Kandelia obovata*, exhibiting high antagonistic activity against *R. solanacearum* in peanuts.
*R. solanacearum*: Isolated, purified, identified, and preserved from naturally occurring peanut disease nurseries.
Preparation of antagonistic bacterial agent: A single colony of *Bacillus velezensis* M03 was picked and placed in NB medium, cultured with shaking at 28°C and 180 r/min for 48 h. Subsequently, the bacterial suspension was centrifuged at 8000 r/min and 4°C for 10 min, and the supernatant was discarded. The collected bacterial pellet was resuspended in sterile deionized water, this process was repeated 3 times, and the concentration of the bacterial suspension was adjusted to 1.0×10^8^ CFU/mL, which was the antagonistic bacterial agent.
Preparation of *Ralstonia solanacearum* suspension: *R. solanacearum* was streaked onto TTC medium containing 1% 2,3,5-triphenyltetrazolium chloride and incubated at 28 °C for 48 h. Typical single colonies with a pink center and milky white edges were picked and placed in NB medium, incubated at 30 °C and 180 r/min for 48 h with shaking. The bacterial concentration was adjusted to 1.0 × 10^8^ CFU/mL to obtain the *R. solanacearum* suspension.

### Experimental methods

300 g of cultivation substrate was filled into 10 cm × 10 cm pots. One seedling per pot was planted using plump, healthy peanut seeds. When the seedlings reached the three-leaf stage, uniformly growing peanut seedlings were selected for the experiment. The treatment groups are as follows:

CK group: Sterile ddH_2_O (20 mL/plant) was applied via root drenching;
Bv group: Antagonistic bacterial inoculant (20 mL/plant) was applied via root drenching;
Rs group: Five punctures were made with a needle at the first tiller of the peanut main stem, followed by 20 mL of Ralstonia solanacearum solution;
Bv+Rs group: A combination treatment of *R. solanacearum* and antagonistic bacteria. Antagonistic bacterial inoculant was first inoculated via root drenching. 2 d after inoculation, *R. solanacearum* suspension was inoculated via puncture, and this was recorded as 0 d of the treatment. Sterile syringes were used for both inoculant and bacterial suspension application.

Each treatment was set with 6 pots, and sample collection was carried out on the 3rd d and 10th d after treatment, respectively. Add “_1” after the code to represent the 3rd d, and “_2” to represent the 10th d.

The treatment groups and their codes are shown in detail in **Table 1**.

**Table 1:** Processing group and number.

No	Group	Group description	3 d	10 d
1	CK	ddH_2_O	CK_1	CK_2
2	Bv	M03 bacterial agent treatment only	Bv_1	Bv_2
3	Rs	Treatment with Ralstonia solanacearum only	Rs_1	Rs_2
4	Bv+Rs	Inoculate with M03 followed by inoculation with Ralstonia solanacearum	Bv+Rs_1	Bv+Rs_2

Bacterial wilt disease incidence was investigated on 3 d (first round of sampling) and 10 d (second round of sampling) after inoculation with *R. solanacearum*. Peanut disease incidence and disease index were statistically analyzed. Simultaneously, peanut leaves and rhizosphere soil samples were collected from each treatment group to determine peanut leaf enzyme activity, soil enzyme activity, soil chemical properties, and for high-throughput sequencing.

The specific steps for rhizosphere soil collection are as follows: The plant was removed from the soil, and large clumps of attached soil were gently shaken off. The roots were placed in a sterile self-sealing bag and gently tapped and shaken to dislodge the attached soil into the bag; this is the rhizosphere soil.

A portion of the rhizosphere soil samples was collected from each treatment group. One portion was stored at 4°C for soil enzyme activity determination, another portion was flash-frozen in liquid nitrogen and stored at -80°C for subsequent DNA extraction and analysis. The remaining samples were air-dried at room temperature for soil chemical property determination. One biological replicate consisted of one pot, with three replicate pots per treatment used for analysis.

Incidence and severity levels of peanut bacterial wilt.

According to the proportion of diseased leaves and the degree of plant wilting (Yilma et al., 2025), the peanut disease grade was divided into 5 grades (see **Supplementary Table 1**).

The formula for calculating the incidence rate is Formula 1, as follows: The formula for calculating the incidence rate is Equation 1, as follows:

(1) $$\text{Incidence rate}=\frac{N_{1}}{N_{0}}\times 100\%$$

Note: N1 is the number of infected plants, and N0 is the total number of plants.

The formula for calculating the disease index is Equation 2, as follows:

(2) $$\;\text{Disease index}=\frac{\sum (N_{i}\times {\text{n}}_{i})}{N_{0}\times {\text{n}}_{\max}}\times 100$$

Note: Ni is the number of infected plants at a certain disease level. ni is the corresponding disease level. N0 is the total number of plants. nmax is the highest disease level value.

### Leaf enzyme activity

The activities of catalase (CAT) in peanut leaves were determined using the ammonium molybdate method (Lian et al., 2023).

Peroxidase (POD) activity in peanut leaves was measured by the guaiacol method (Hu et al., 2023).

Superoxide dismutase (SOD) activity in peanut leaves was determined using a xanthine/xanthine oxidase reaction system that generates superoxide anions (O_2_^-^), which react with WST-1 to form water-soluble yellow formazan (Qiu et al., 2023).

Malondialdehyde (MDA) content in peanut leaves was measured based on its condensation with thiobarbituric acid (TBA) under acidic and high-temperature conditions (Xu et al., 2023).

Soluble sugar content in peanut leaves was determined by the anthrone colorimetric method (Du et al., 2023).

### Soil chemical properties

Available phosphorus (AP) was extracted using the HClO_4_-H_2_SO_4_ digestion method. Available phosphorus and total phosphorus (TP) contents were determined using a SAN++ continuous flow analyzer (SKalar, Netherlands). Available potassium (AK) was extracted using 1.0 mol·L^-^¹ CH_3_COONH_4_ solution. Its content was determined using a PinAAcle 900H flame atomic absorption spectrometer (PERKINELMER, USA). Soil samples were pretreated with 0.5 mol·L^-^¹ HCl to remove inorganic carbonates. Ammonium nitrogen (NH_4_^+^-N) and nitrate nitrogen (NO_3_^-^-N) contents were determined using the indophenol blue colorimetric method and the nitrosalicylic acid colorimetric method. Available sulfur (AS) content was determined using the barium sulfate turbidimetric method (Gu et al., 2019; Liu et al., 2024).

### Soil enzyme activity

Soil urease activity was determined using the indophenol blue colorimetric method (Guo et al., 2012). Soil acid phosphatase activity was determined using the phenol colorimetric method (Hou et al., 2020). Soil leucine aminopeptidase activity was determined using the p-nitroaniline colorimetric method (Saiya-Cork et al., 2002).

### DNA extraction and sequencing

Accurately weigh 0.5 g of soil sample and extract soil DNA using the E.Z.N.A.^®^ Soil DNA Kit (Omega, USA). After the DNA quality meets the standards (total amount not less than 15 μg, concentration not less than 50 ng·μL^-^¹), amplify the 16S rRNA V3~V4 variable region of each sample using the universal bacterial primers 338F (5′-ACTCCTACGGGAGGCAGCAG-3′) and 806R (5′- GGACTACHVGGGTWTCTAAT-3′). Amplify the variable region ITS1 between the 18S rRNA and 5.8S rRNA genes and the variable region ITS2 between the 5.8S rRNA and 28S rRNA genes using the universal fungal primers ITS1 (5′-TCCGTAGGTG AACCTGCGG-3′) and ITS2 (5′-GCTGCGTTCTTCATCGATGC-3′). The total volume of the PCR system was 50 μL, containing 5 μL of 10×Buffer, 5 μL of 2 mmol·L^-^¹ dNTPs, 1 μL of DNA polymerase, 1.5 μL each of forward and reverse primers, 50 ng of DNA template, and double-distilled water to make up the volume. The PCR program was as follows: 94 °C pre-denaturation for 10 min, 94 °C denaturation for 30 s, 56 °C annealing for 30 s, 72 °C extension for 30 s, for 35 cycles; followed by a 72 °C stable extension for 10 min. Each sample was repeated in triplicate. No template negative control was included.

The PCR products were quality-tested using the QuantiFluor™-ST blue fluorescence quantitative PCR system (Promega, USA). After passing the quality test, the products were sequenced on the Illumina Miseq platform (Shanghai Meiji Biotechnology Co., Ltd.).

### Soil microbial community analyses

The Flash v1.2.11 software was used for paired-end sequence assembly to generate complete gene sequences (MagočSalzberg, 2011). RDP Classifier v2.13 software was used for OTU (operational taxonomic unit) clustering annotation to obtain sequence classification information (Wang et al., 2007). Usearch v11 software was used to count the number of OTUs and calculate the relative abundance of microorganisms (Edgar, 2010). Qiime v1.9.1 software was used to generate a table of absolute abundance of microorganisms. Principal component analysis (FactoMineR method) based on the number of OTUs was performed using the vegan package in R v4.1.3 software, and the results were visualized using the ggplot2 v3.3.3 package in R v4.1.3 software (Warton et al., 2012). Spearman correlation analysis was performed using the stats package in R v3.6.3 software, and the results were visualized using the pheatmap package in R v3.6.3.

Niche breadth indices for each species were calculated based on OTU absolute abundance using the spaa package in R v4.2.3. Species occurrence frequencies were simulated using randomization rearrangement via the EcolUtils package in R v4.2.3. Species with niche breadth indices exceeding the upper limit of the 95% confidence interval were defined as generalists, and those below the lower limit were defined as specialists (Zhang et al., 2018). FAPROTAX v1.2.1 was used for bacterial community functional prediction. FUNGuild v1.0 was used for functional prediction of the fungal community. The ggClusterNet package in R v4.2.3 software was used to calculate topological characteristic indices (Ma et al., 2016), and Gephi v0.9.7 was used for co-occurrence network visualization (|r| > 0.8, *p*< 0.01). The linkET package (v0.0.7.4) in R v4.2.3 software was used to perform Mantel test calculation (*p*< 0.05), and visualization was conducted by ggplot2 (v3.3.3). SPSS 23.0 was used for significance test of all data.

### Statistical analysis

Data preprocessing was performed using Excel 2019 and SPSS 23.0, analysis of variance and *post-hoc* tests were conducted on peanut leaf antioxidant enzyme activity, soil chemistry, and enzyme activity indices (using One-way ANOVA + Fisher LSD, *p*< 0.05).

## Results

### Effects of *B. velezensis* M03 on peanut disease incidence and disease index

As shown in **Supplementary Table 2**, no cases developed in either the CK or Bv groups during the entire experiment. In the Rs group, the incidence rate reached 50.00% on 3 d, with a disease index of 16.67. On 10 d, the incidence rate rose to 100.00%, and the disease index reached 54.17. In the Bv+Rs group, the incidence rate on 3 d was 16.67%, representing a 66.67% inhibition rate compared to the Rs group; the disease index was 4.17, representing a 74.99% inhibition rate compared to the Rs group’s disease index of 16.67. On 10 d, both the incidence rate and disease index in the Bv+Rs group increased (reaching 50.00% and 16.67, respectively), compared to the Rs group, its incidence rate inhibition rate was 50.00%, and its disease index inhibition rate was 69.23%.

### Effects of *B. velezensis* M03 on stress-related physiological indicators of peanut plants

#### Changes in antioxidant enzyme activity in peanut leaves

**Figure 1:**
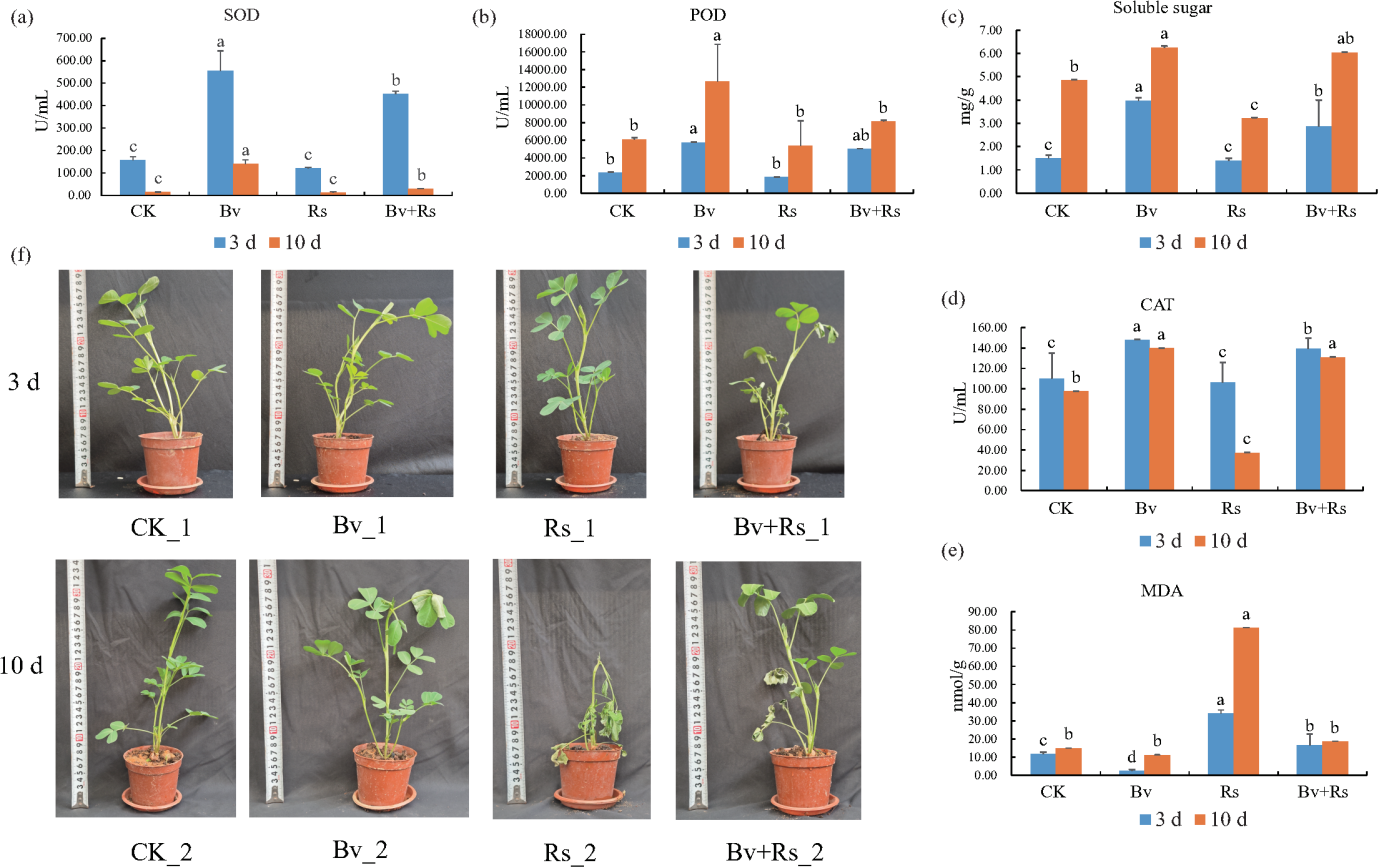
Changes in antioxidant enzyme activity, oxidative damage, and osmotic regulation substances in peanut leaves. **(a)** Superoxide dismutase activity; **(b)** Peroxidase activity; **(c)** Soluble sugar content; **(d)** Catalase activity; **(e)** Malondialdehyde content; **(f)** Representative images of peanut seedling growth under CK, Bv, Rs, and Bv+RS treatments at 3 d and 10 d. Different lowercase letters indicate significant differences among CK, Bv, Rs, and Bv+RS treatments at the same sampling day (*p*< 0.05).

Regarding SOD enzyme activity (**Figure 1**, **Supplementary Table 3**), Bv_1 had the highest activity (554.04 ± 89.51 U/mL), which was higher than other sample; Bv_2 sample maintained the highest activity (141.56 ± 17.45 U/mL), which was significantly higher than the control sample CK_2 (14.96 ± 0.86 U/mL). POD enzyme activities showed similar trends, with the Bv_1 sample exhibiting significantly higher activity (5741 ± 56.43 U/mL), which was 3.15 times that of the Rs_1 sample (1820.67 ± 35.85 U/mL). The POD activity of the combination treatment sample (Bv+Rs_1) was 5025.33 ± 31.66 U/mL. The Bv_2 sample maintained a significant advantage in POD activity (12658.33 ± 4186.94 U/mL), exceeding the Rs_2 sample (5357.33 ± 2857.73 U/mL) by 136%. The POD activity of the combination treatment sample (Bv+Rs_2) reached 64.2% of that of the Bv_2 sample (8134.00 ± 129.64 U/mL). CAT enzyme activity was highest in the Bv_1 sample (147.78 ± 0.59 U/mL), a 34.3% increase compared to the control sample CK_1 (110.01 ± 25.02 U/mL). CAT enzyme activity in the Rs_2 sample was severely inhibited (37.16 ± 0.31 U/mL), only 26.6% of that in the Bv_2 sample. The combined treatment sample (Bv+Rs_2) showed CAT enzyme activity of 130.98 ± 0.44 U/mL, close to the level of the Bv_2 sample. In summary, a common characteristic of all samples was that the SOD, POD, and CAT enzyme activities in peanut leaves were significantly higher than those in the control after treatment with *B. velezensis* M03.

#### Oxidative damage and changes in osmotic regulators in peanut leaves

Regarding malondialdehyde (MDA) content (**Figure 1**, **Supplementary Table 3**), Rs_1 sample had the highest MDA content (34.05 ± 1.88 nmol/mL), which was 14.6 times that of Bv_1 (2.34 ± 0.90 nmol/mL). The MDA content of Rs_2 sample further increased to 81.12 ± 0.17 nmol/mL, while that of Bv+Rs_2 sample decreased significantly to 18.56 ± 0.18 nmol/mL, a decrease of 77.1%, but this content was still higher than that of Bv_2 (11.10 ± 0.14 nmol/mL). The soluble sugar content determination results showed that the Bv group had the highest content in both samplings, at 3.97 ± 0.12 mg/g and 6.25 ± 0.06 mg/g, respectively. The soluble sugar content of the Bv+Rs_2 sample reached 6.03 mg/g, which was significantly higher than that of Rs_2 (3.21 ± 0.04 mg/g).

### Effects of *B. velezensis* M03 on soil chemical properties

Regarding ammonium nitrogen content (**Supplementary Table 4**), Rs_1 sample had the highest content (15.99 ± 1.83 μg/mL), significantly higher than CK_1 (13.01 ± 0.52 μg/mL, *p*< 0.05). Rs_2 sample still maintained the highest ammonium nitrogen content (13.27 ± 0.10 μg/mL), an increase of 34.6% compared to CK_2 (9.86 ± 0.18 μg/mL). However, Bv+Rs_2 sample significantly decreased to 12.45 ± 0.27 μg/mL, a decrease of 6.4% compared to Rs_2 (*p*< 0.05).

Nitrate nitrogen content showed inter-group differences, with Bv_1 sample exhibiting the highest content (6.00 ± 0.34 μg/mL), which was 3.5 times that of Rs_1 (1.70 ± 0.29 μg/mL) (*p*< 0.05). Bv_2 sample maintained its advantage in nitrate nitrogen content (5.44 ± 0.02 μg/mL). Furthermore, the nitrate nitrogen content in the Bv+Rs group was significantly increased, more than doubling compared to the Rs group. For example, Rs_2 sample was 1.21 μg/mL, while Bv+Rs_2 reached 4.95 μg/mL (*p*< 0.05).

Regarding available phosphorus (**Supplementary Table 4**), no significant differences were observed between the two groups. However, the AP values of both the Bv and Bv+Rs groups were relatively high (e.g., Bv_1: 5.00 μmol/mL, Bv+Rs_1: 5.16 μmol/mL; Bv_2: 4.65 μmol/mL, Bv+Rs_2: 4.73 μmol/mL). The AP value of the Rs group was consistently the lowest (e.g., Rs_1: 2.14 ± 0.39 μmol/mL; Rs_2: 1.72 ± 0.14 μmol/mL). In contrast, regarding total phosphorus content, Bv_1 sample (296.12 ± 17.15 μg/g) and Bv+Rs_1 sample (282.59 ± 13.17 μg/g) were significantly higher than Rs_1 (176.97 ± 16.97 μg/g, *p*< 0.05).

The TP content of Bv_2 sample remained the highest (275.92 ± 11.78 μg/g), while Rs_2 further decreased to 155.39 ± 5.81 μg/g, only 56.3% of that of Bv_2. Further analysis of available potassium (AK) showed that the content of Bv_2 sample (0.07 ± 0.00 mg/kg) was significantly higher, 1.75 times that of CK_2 sample (0.04 ± 0.00 mg/kg) (*p*< 0.05). Simultaneously, the AK value of the combined treatment Bv+Rs_2 sample (0.06 ± 0.00 mg/kg) was also significantly higher than that of Rs_2 (0.04 ± 0.00 mg/kg, *p*< 0.05).

Regarding available sulfur (**Supplementary Table 4**), Bv_1 sample (238.50 ± 13.05 mg/kg) was significantly the highest, its value was 4.8 times that of Rs_1 (49.50 ± 0.89 mg/kg) (*p*< 0.05). The combined treatment of Bv+Rs_2 sample increased the AS value to 196.83 ± 6.86 mg/kg, which was significantly higher than Rs_2 (46.83 ± 0.58 mg/kg) (*p*< 0.05).

### Effects of *B. velezensis* M03 on soil enzyme activity

Soil phosphatases (**Supplementary Table 5**) showed the highest activity in Bv_1 sample (282,400.25 ± 1,311.38 U/g), significantly higher than the CK_1 sample (252,921.40 ± 748.23 U/g, *p<* 0.05). Rs_1 sample showed the lowest activity (250,898.19 ± 1,277.48 U/g). Bv_2 (245,265.74 ± 1,210.00 U/g) and the Bv+Rs_2 sample (243,933.88 ± 900.91 U/g) jointly maintained the highest activity, representing a 26.6% increase over Rs_2 (193,699.34 ± 1,462.06 U/g) (*p*< 0.05).

Soil urease activity was highest in Bv_1 sample (7,167.19 ± 27.21 U/g), which was 3.93 times that of Rs_1 (1,823.20 ± 8.21 U/g) (*p<* 0.05). Bv_2 sample (5,952.50 ± 45.64 U/g) still maintained the highest activity. The urease activity of the Bv+Rs_2 sample was significantly increased (from 1,845.00 → 4,310.70 U/g, *p<* 0.05), while Rs_2 remained the lowest (1,845.00 ± 3.55 U/g).

The activity of leucine aminopeptidase in soil was highest in Bv_1 sample (8.96 ± 0.34 U/g), which was 3.36 times that of CK_1 (2.67 ± 0.01 U/g) (*p*< 0.05). Rs_1 sample showed the lowest activity, appearing to be severely suppressed (0.09 ± 0.07 U/g), less than 1% of the activity in the Bv group. The activity in Bv+Rs_2 sample increased from 0.06 U/g in Rs_2 to 1.10 U/g (a 17.3-fold increase, *p<* 0.05). Bv_2 sample (1.49 ± 0.21 U/g) was still significantly higher than other treatments (*p*< 0.05).

**Figure 2:**
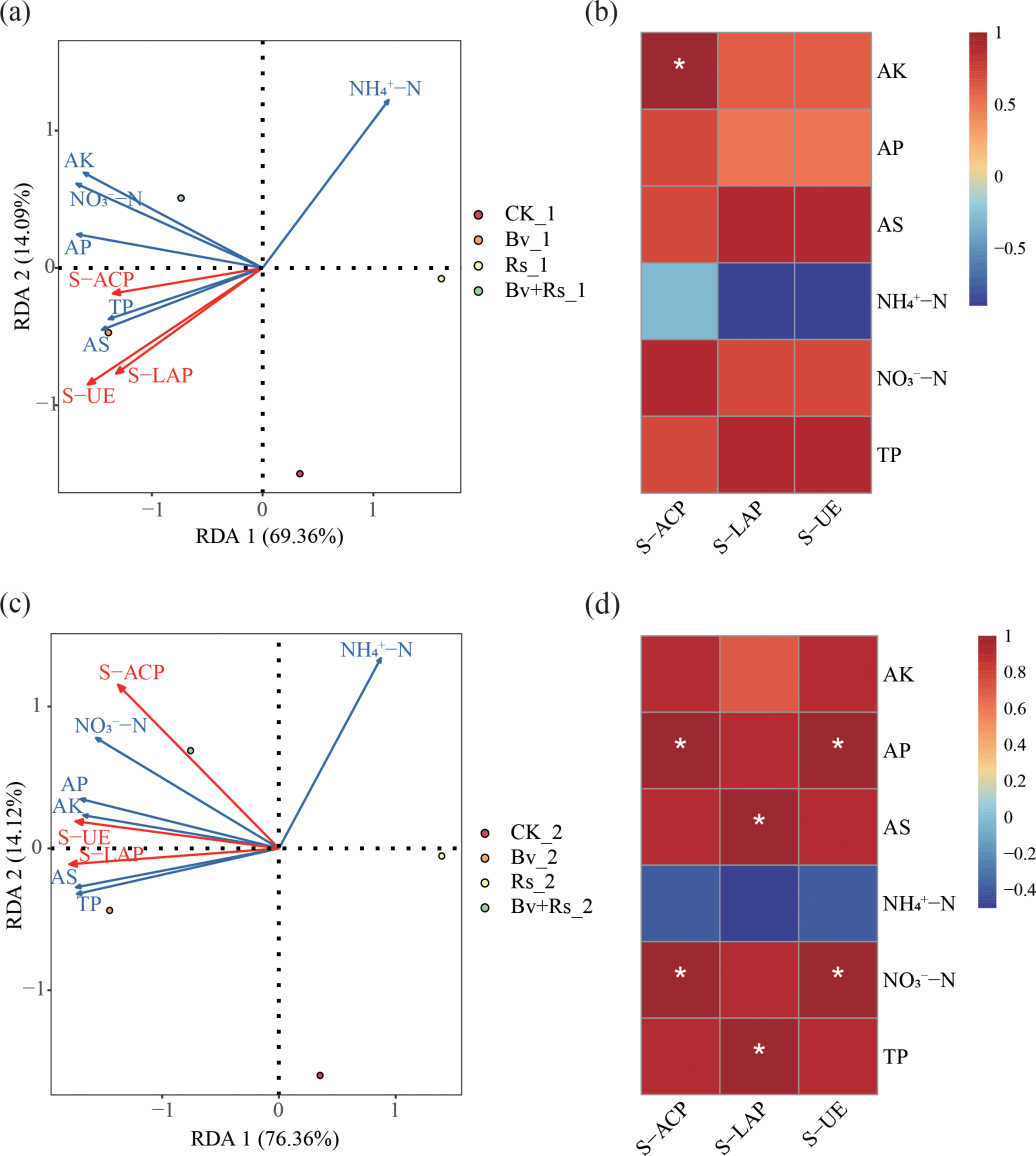
Principal component analysis and correlation of soil chemical indicators and enzyme activity. **(a)** Redundancy analysis of soil chemical properties and soil enzyme activities at 3 d; **(c)** Redundancy analysis of soil chemical properties and soil enzyme activities at 10 d; **(b)** Correlation heatmap of soil chemical properties and soil enzyme activities at 3 d; **(d)** Correlation heatmap of soil chemical properties and soil enzyme activities at 10 d. “*” indicates a significant correlation between enzyme activity and corresponding chemical property (*p*< 0.05).

We investigated the correlation between soil enzyme activity and chemical properties using principal component analysis (RDA) and Spearman correlation analysis. The results (**Figure 2**) showed that principal components 1 and 2 explained 83.45% of the data (3 d, first round of sampling). The activities of the three soil enzymes were positively correlated with Bv_1 sample. Except for ammonium nitrogen, all other soil chemical indicators were positively correlated with Bv_1 and Bv+Rs_1 sample. However, only phosphatase showed a significant positive correlation with available potassium (*p<* 0.05). Principal components 1 and 2 explained 90.48% of the data (10 d, Second round of sampling). The activities of the three soil enzymes were positively correlated with Bv_2 and Bv+Rs_2 sample. Similar to the first round of sampling (3 d), except for ammonium nitrogen, all other soil chemical indicators were positively correlated with Bv_2 and Bv+Rs_2 sample. Phosphatase and urease were significantly positively correlated with available phosphorus and nitrate nitrogen. Peptidase was significantly positively correlated with available sulfur and total phosphorus (*p<* 0.05).

### Impact of *B. velezensis* M03 on soil microecology

#### Changes in soil microbial species

High-throughput sequencing revealed that the increase in detectable microbial species tended to level off as the gene sequence in each sample increased, indicating that the detected microbial species in the sample could represent the composition of the microbial community in that sample. In the statistical analysis of detectable microorganisms in each sample, the changes from phylum to family were relatively small. However, at the genus level (**Supplementary Table 6**), the bacterial counts in CK_1 and CK_2 samples were highly stable. Bv_1 and Bv_2 samples showed significant differences, with Bv_1 sample having the lowest bacterial count (400) and Bv_2 reaching a maximum of 453. The Bv+Rs group showed the opposite trend to the Bv group in both measurements. Regarding fungi, the control group was also relatively stable with little difference. Similar to bacteria, the Bv group showed the lowest count (100) in Bv_1 sample and a maximum of 153 in Bv_2 sample; the Bv+Rs group also showed the opposite trend in both measurements. The lack of significant dynamic changes in the control group indicates that the microbial community of the control soil was stable. The Bv group increased the variety of bacteria and fungi in the community.

#### Changes in soil microbial diversity

The bacterial Shannon index revealed that the Bv+Rs_1 group (7.90) was significantly higher than the Rs_1 group (7.36). On day 10, the Bv_2 group (8.16) was significantly higher than the CK_2 group (7.75) and the Bv+Rs_2 group (7.44).The bacterial Chao1 index on day 10 showed that the Bv_2 group (2892.14) and Rs_2 group (2914.71) were significantly higher than the Bv+Rs_2 group (2282.04). For fungal communities, the Shannon index on day 3 was highest in the Bv+Rs_1 group (3.99) and CK_1 group (3.88), which were significantly higher than the Bv_1 group (3.46). On day 10, the Bv+Rs_2 group (3.11) was significantly lower than the Bv_2 group (3.86) and Rs_2 group (3.96).The fungal Chao1 index was highest in the Bv+Rs_1 group (325.11) on day 3. On day 10, the Rs_2 group (399.47) and Bv_2 group (393.33) were significantly higher than the Bv+Rs_2 group (280.18), as shown in **Supplementary Table 7**. Based on principal component analysis (**Supplementary Figure 1**), for bacterial communities (R = 0.52, *p* = 0.001), the first principal component (PC1) and second principal component (PC2) explained 44.73% and 16.39% of the total variation, respectively. On day 3, all treatments were relatively concentrated along the PC1 axis, but the Bv+Rs_1 group and Rs_1 group showed a separation trend along the PC2 axis. On day 10, the dispersion degree of all treatments along the PC1 axis increased significantly. The Bv_2 and Rs_2 groups were far from the CK_2 group, and the Bv+Rs_2 group was between the CK_2 and Rs_2 groups. For fungal communities (R = 0.18, *p* = 0.014), PC1 and PC2 explained 39.22% and 36.56% of the total variation, respectively. The CK_1 group was located at the positive end of PC1, the Bv_1 and Rs_1 groups were on the negative axis, and the Bv+Rs_1 group was between them. The CK_2, Bv_2, and Rs_2 groups were all concentrated on the negative axis of PC1, while the Bv+Rs_2 group was separately located at the positive end, showing obvious separation from the other treatments.

### Distribution of dominant microorganisms

**Figure 3:**
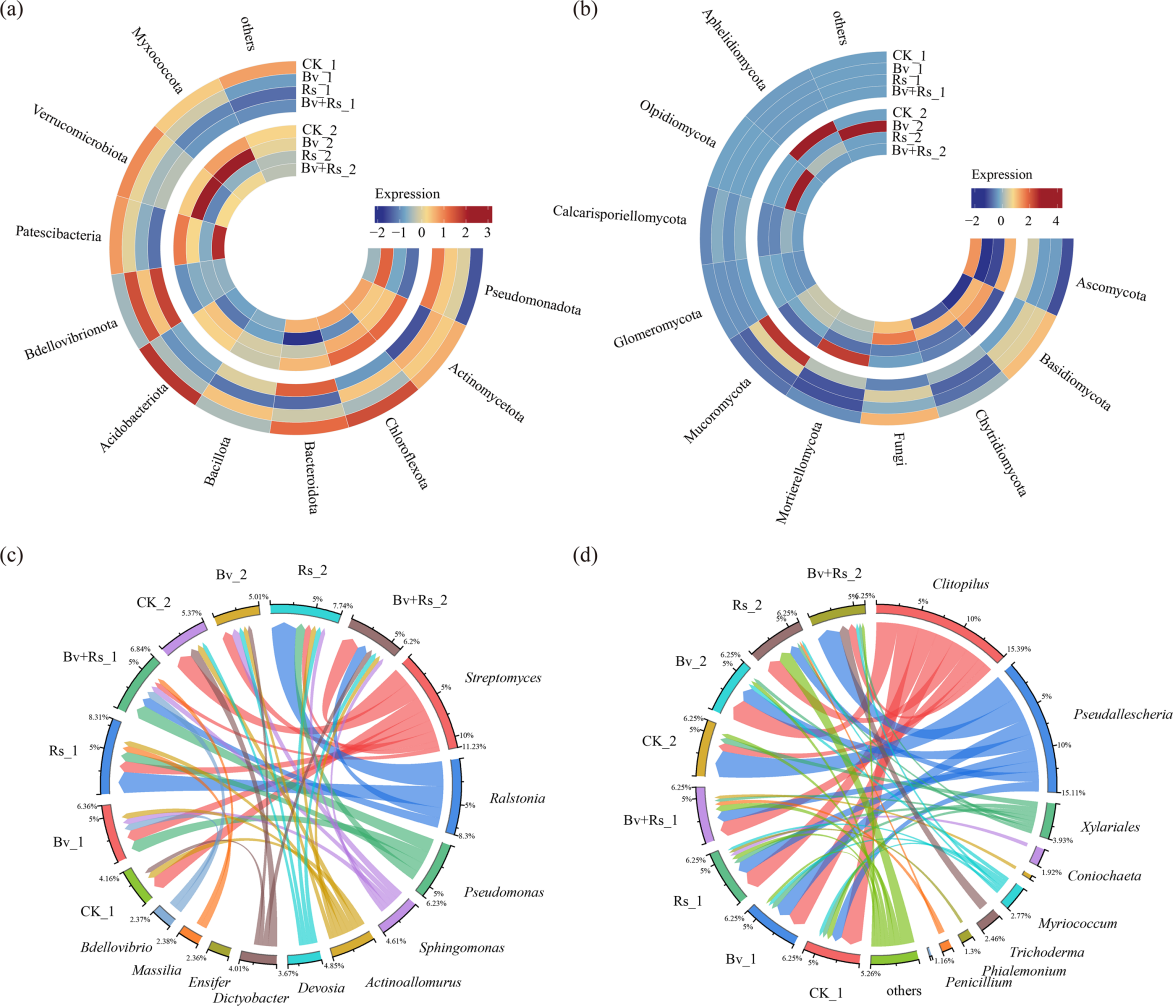
The impact of *Bacillus velezensis* M03 on dominant species at the phylum and genus level in soil microorganisms. **(a)** Heatmap of the top 10 relative abundances of bacterial communities at the phylum level at 3 d; **(b)** Heatmap of fungal relative abundances at the phylum level at 10 d; **(c)** Circos plot of the top 10 bacterial communities at the genus level at 3 d; **(d)** Circos plot of the top 10 bacterial communities at the genus level at 10 d.

Among the relative abundances of bacteria Phylum (**Supplementary Table 8**, **Figure 3**), Pseudomonadota (Bv+Rs_1) had the highest abundance (51.98%) in the first round of samples. The CK group (27.31%) had only about half that. In the second round of samples, Rs_2 sample increased to 54.14%, the highest abundance among all treatments. Bv_2 sample decreased to 34.39% (a decrease of 16.7% compared to Bv_1). The relative abundance of Actinomycetota (Bv+Rs_2) was higher than that of Rs_2 (20.50%) in the second round. The relative abundance of Chloroflexota decreased significantly in the Rs group (from 13.30% in Rs_1 to 6.53% in Rs_2, a decrease of 50.9%). In the CK group, it maintained a consistently high abundance (CK_1: 18.72%; CK_2: 17.21%).

Among the relative abundances of key bacterial genera (**Supplementary Table 9**, **Figure 3**), *Streptomyces* showed a 24.1% increase in relative abundance in CK2 sample (0.129%) compared to CK1 (0.104%). This increase was also observed in the Bv+Rs groups (Bv+Rs1: 0.035%, Bv+Rs2 sample: 0.130%, an increase of 269%). However, its abundance in the Rs groups remained at a low level (Rs1: 0.082%; Rs2: 0.046%). *Ralstonia* exhibited abnormal proliferation in the Rs groups: the relative abundance of Rs1 was 0.159% (116 times that of Bv1), and Rs2: 0.185% (848 times that of CK2). Significant inhibition was observed in the Bv+Rs groups: abundance decreased by 55.9% (from 0.159% in Bv+Rs1 to 0.072% in Bv+Rs2).

At the fungal Phylum level (**Supplementary Table 10**, **Figure 3**), Ascomycota was significantly enriched in both Bv+Rs_1 and Bv+Rs_2 samples, with the highest relative abundance reaching 74.75% (an increase of 19.0% compared to Bv_1), the highest among all treatments. Basidiomycota, however, was highest in CK_1 (49.40%).

At the fungal genus level (**Supplementary Table 11**, **Figure 3**), *Clitopilus* (29.5%) remained the most abundant fungal species in Bv+Rs_1 sample. In Bv+Rs_2 sample treated with both *R. solanacearum* and *B. belye*, *Pseudallescheria* (45.1%) and *Trichoderma* (18.5%) showed a synergistic increase, with their combined relative abundance exceeding 60%. In Rs_2 sample treated only, the proportion of “unclassified bacteria” surged to 26.2%, making Rs_2 sample 3.2 times higher than Rs_1.

While monitoring changes in dominant bacterial communities, we also investigated the relative abundance changes of *B. velezensis* M03 and *R. solanacearum* in each sample. The results (**Supplementary Table 12**) showed that the relative abundance of *R. solanacearum* in both the Bv and Rs treatment groups did not change significantly during the experimental period (*p*< 0.05). Notably, compared to the Rs treatment group (10.00% ± 0.01), the Bv+Rs treatment group (5.00% ± 0.01) significantly reduced the relative abundance of *R. solanacearum*. Furthermore, even though the relative abundance of *B. velezensis* M03 significantly decreased in the Bv+Rs treatment group during the experimental period, the relative abundance of *R. solanacearum* under the same treatment did not increase significantly; instead, it slightly decreased. In contrast, the relative abundance of *R. solanacearum* increased in the Rs treatment group.

### Microbial community niche changes and functional prediction

**Figure 4:**
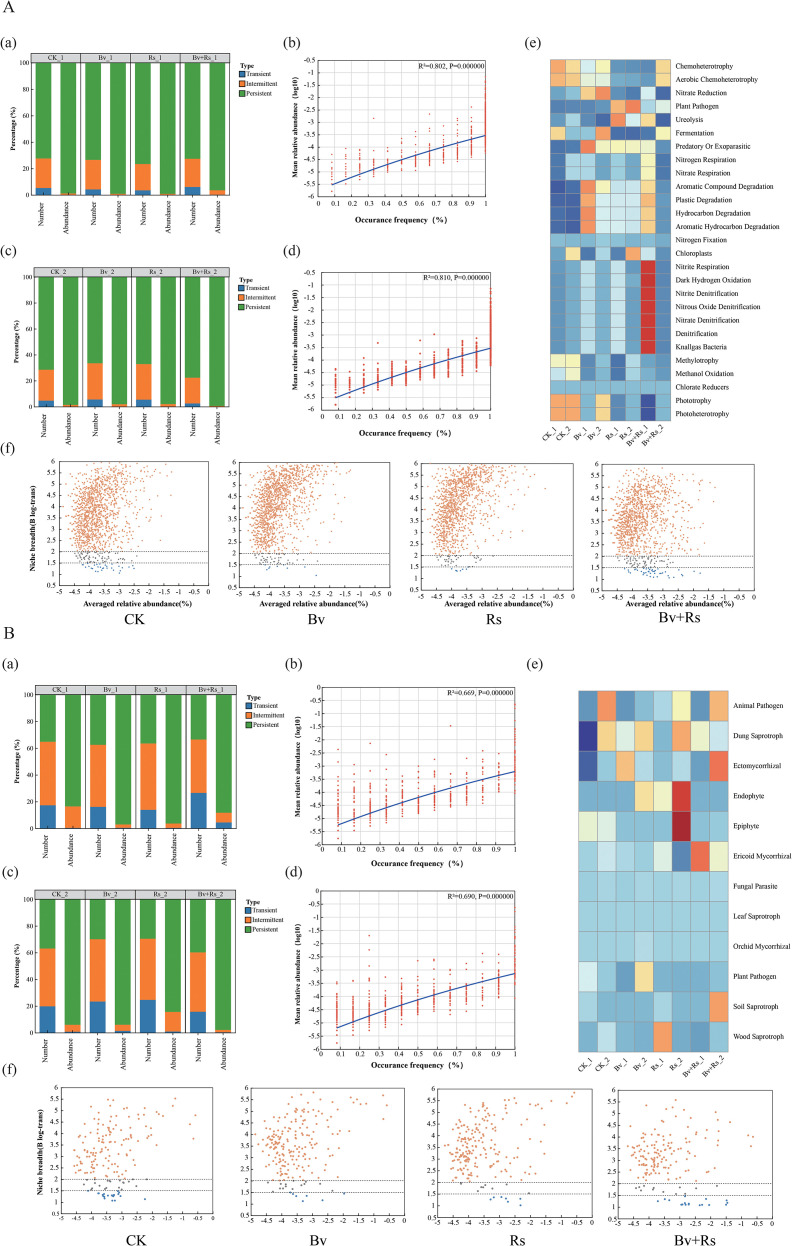
Microbial community niche changes and functional predictions in samples treated with different methods. **(A)** shows the niche changes and functional prediction of the bacterial community, where a is the distribution of transient, intermediate, and persistent bacterial species at 3 d; **(b)** is the distribution of transient, intermediate, and persistent bacterial species at 10 d; **(c)** is the correlation analysis between species prevalence and relative abundance at 3 d; **(d)** is the correlation analysis between species prevalence and relative abundance at 10 d; **(e)** is the functional prediction of CK, Bv, Rs, and Bv+RS treatments at 3 d and 10 d; **(f)** is the niche width at the OTU level of CK, Bv, Rs, and Bv+RS treatments. **(B)** shows the niche changes and functional prediction of the fungal community, where **(a)** is the distribution of transient, intermediate, and persistent fungal species at 3 d; **(b)** is the distribution of transient, intermediate, and persistent fungal species at 10 d; **(c)** is the correlation analysis between species prevalence and relative abundance at 3 d; **(d)** is the correlation analysis between species prevalence and relative abundance at 10 d; **(e)** is the functional prediction of CK, Bv, Rs, and Bv+RS treatments at 3 d and 10 d; **(f)** is the niche width at the OTU level of CK, Bv, Rs, and Bv+RS treatments.

In the bacterial communities of the second round of sampling (**Figure 4**), the correlation between species prevalence and mean relative abundance in the samples decreased (3 d: 0.814, 10 d: 0.801). Specifically, the relative abundance of persistent representative species (core species) increased only in the Bv+Rs treatment group (73.7% → 74.04%). All other samples showed a decrease, with the Rs treatment group showing the largest decrease at 9.98%. Regarding niche changes, generalized species were predominant overall. Compared to the control (CK), the relative abundance of rare species increased in the Bv+Rs treatment group (3.21%) (CK was 1.99%). All other treatments showed a decrease, with the Bv and Rs treatment groups showing the largest and similar decreases. Functionally, relative to the high abundance of plant pathogens expressed in the Rs treatment group, the relative abundance of pathogenic bacteria decreased after application of *B. velezensis* M03 (Bv+Rs group). Meanwhile, Bv treatment did not induce the expression of other plant pathogen functions. Furthermore, application of *B. velezensis* M03 increased the abundance of aromatic hydrocarbon metabolite degradation functions. Notably, Bv and Rs treatments alone had no significant effect on soil nitrogen cycle-related functions. However, the Bv+Rs treatment increased the expression of nitrogen cycle-related functions in the soil.

In terms of fungal communities (**Figure 4**), the correlation between species prevalence and the average relative abundance in the samples also decreased (first round of sampling 3 d: 0.684; second round of sampling 10 d: 0.637). The relative abundance of persistent species (core species) did not change significantly in the CK group. The performance of the Bv+Rs treatment group also increased. There was a decrease in the Bv treatment group and Rs treatment group. In terms of ecological niche, generalization is also the main body. The relative abundance of rare types in the CK group was the highest at 12.29%, followed by the Bv+Rs treatment group at 10.06%. In terms of relative abundance analysis of functional fungi, Endophyte and Epiphyte had the highest abundance in the Rs treatment group.

### Collinear network diagram

**Figure 5:**
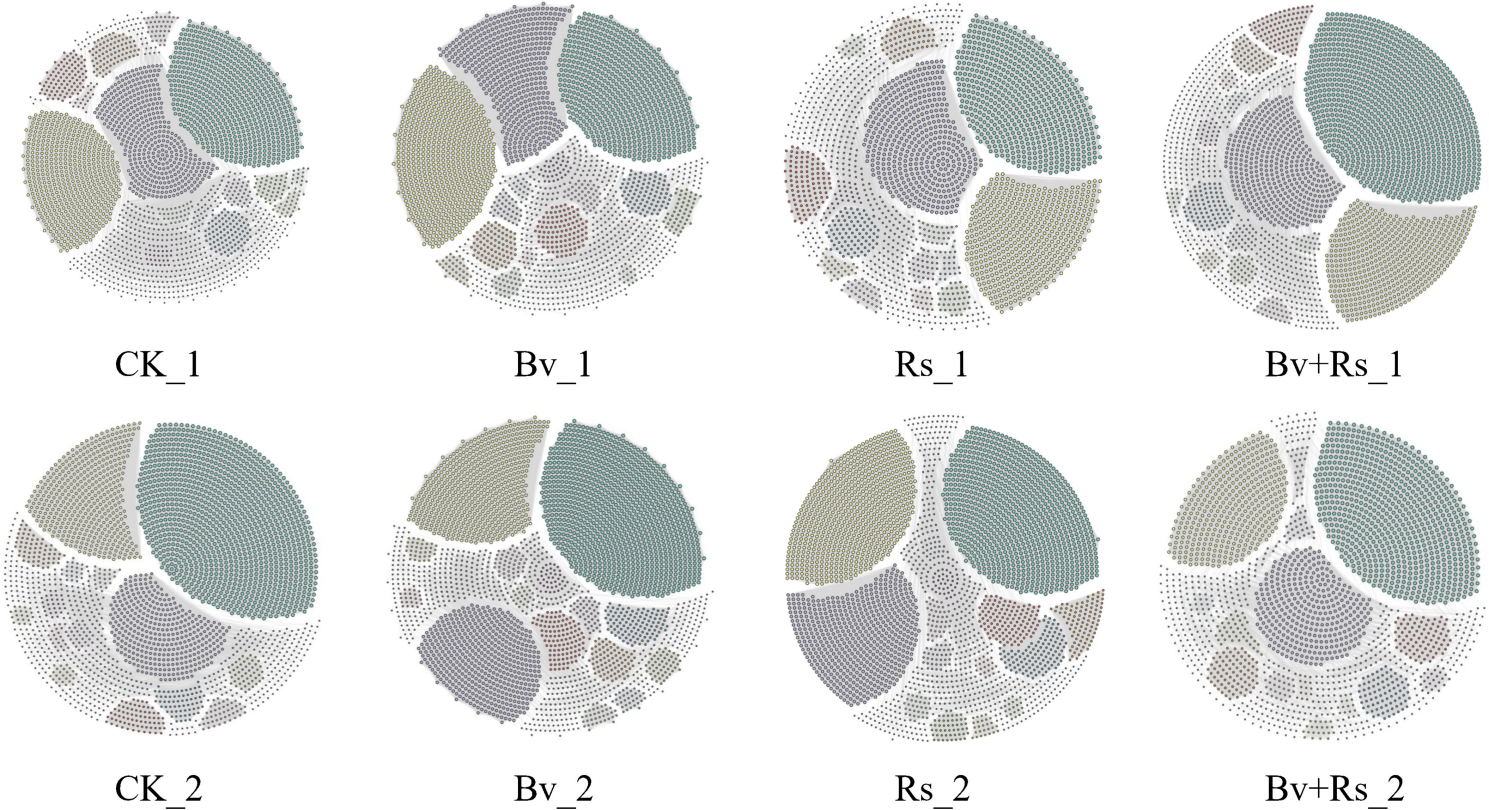
Collinear network diagram. Microbial network of absolute OTU abundance under CK, Bv, Rs, and Bv+Rs treatments.

Regarding bacterial communities (**Supplementary Table 13**, **Figure 5**), the CK treatment group constructed a network with the most edges (7412) and the highest average degree (74.49). The Rs treatment group was the most conservative, with the lowest number of edges (6443.5) and average degree (64.76). Notably, the Bv+Rs treatment group largely recovered the connections filtered out by Rs, and its network size (7195.5 edges, 72.65 average degree) was very close to that of the CK treatment group. Positive correlations were slightly dominant, fluctuating between 49.89% and 54.50%. The Bv+Rs treatment group detected the highest proportion of positively correlated edges (54.50%). In terms of relative modularity, the CK group was the lowest. Although the Rs treatment group showed some improvement, it was still lower than the improvement effect after the application of *B. velezensis* M03.

Analysis of the fungal community co-occurrence network topology showed that the Bv+Rs treatment group had the densest network structure (**Supplementary Table 14**, **Figure 5**), with the highest number of edges (5241.5) and average degree (52.42). The Bv treatment group was the sparsest (4616 edges, average degree 46.16), and the fungal networks exhibited a very high proportion of positive correlations (57.83% - 73.33%), far exceeding that of the bacterial communities. Specifically, the Bv+Rs treatment group had a positive edge ratio as high as 73.33%. Regarding relative modularity, the differences among the treatments were relatively small (6.90 - 7.66).

M03 and *R. solanacearum* exhibit significant ecotypic differences in shaping the functional potential of soil microorganisms. Furthermore, they indirectly influence the distribution of microbial functional genes by regulating soil chemical properties and enzyme activity. From the perspective of soil chemical and enzymatic characteristics, the two bacteria show specificity in their impact on the soil microenvironment. Specifically, in the presence of *B. velezensis*, the soil contains relatively higher levels of NH_4_^+^-N and TP, as well as urease (S-UE) and leucine aminopeptidase (S-LAP) activities.

**Figure 6:**
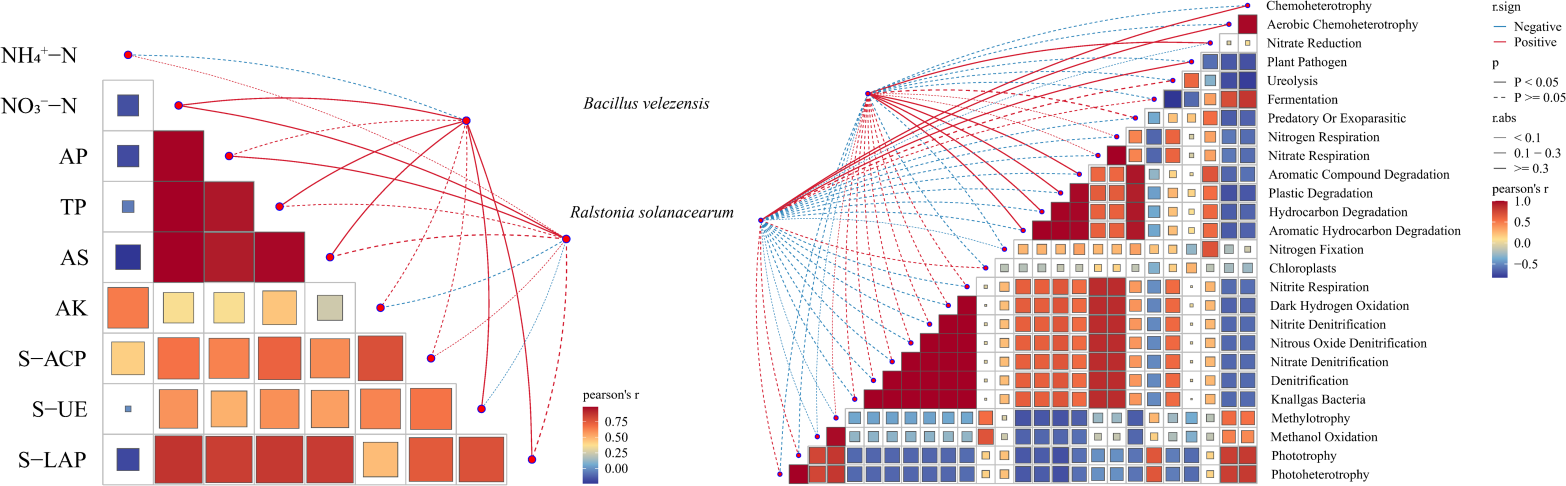
Correlation between *Bacillus velezensis* M03 and *Ralstonia solanacearum* and soil microecology and bacterial function. Note: Mantel test (*p*< 0.05) between the antagonistic bacterium *Bacillus velezensis*, the pathogenic bacterium *Ralstonia solanacearum*, microbial functional prediction, soil chemical properties, and soil enzyme activities, visualized as a collinearity network diagram.

Further functional gene analysis of microbial functional responses revealed significant differences in the abundance of genes involved in carbon cycling (e.g., *Cellulolysis*, *Xylanolysis*), nitrogen cycling (e.g., *Nitrogen Fixation*, *Denitrification*), phosphorus cycling (e.g., *Phosphorus Solubilization*), and biological interactions (e.g., *Antibiotic Synthesis*, *Metal Resistance*) in the Bv+Rs treatment group (**Figure 6**). Under the influence of *B. velezensis*, the abundance of genes related to nitrogen fixation, cellulose decomposition, and antibiotic synthesis was higher. In soils associated with *R. solanacearum*, genes related to nitrate reduction and metal resistance, which are associated with pathogen adaptation or infection, are more enriched.

## Discussion

### Effects of *B. velezensis* M03 on peanut stress resistance

#### *B. velezensis* M03 alleviates membrane lipid peroxidation by increasing antioxidant enzyme activity

Studies have shown that *B. velezensis* M03 significantly increases the activities of SOD, POD, and CAT in peanuts while decreasing MDA content. This indicates that M03 can reduce membrane lipid peroxidation damage by enhancing ROS scavenging capacity. This result is consistent with the report by Li et al. in the tomato-*B. velezensis* system (Li et al., 2025). The mechanism may be related to extracellular polysaccharides and volatile organic compounds (VOCs) secreted by antagonistic bacteria. These compounds may activate plants to up-regulate the expression of antioxidant-related genes (Zhou et al., 2024). Notably, in the second round of sampling, Rs treatment resulted in only 26.6% of CAT activity compared to Bv treatment. Such differences between treatments are similar to those observed in studies with co-inoculation of Pseudomonas fluorescens and the pathogen *Fusarium oxysporum* (Sun et al., 2022).

#### *B. velezensis* M03 enhances peanut stress resistance by restoring redox balance and sugar metabolism function

Although *R. solanacearum* (Rs) treatment caused severe oxidative damage, *B. velezensis* and *R. solanacearum* (Bv+Rs) treatment restored POD and CAT activities to 64%-97% of those in Bv treatment, while reducing MDA content by 77%, demonstrating a significant “repair effect.” This aligns with the “stress-recovery” model proposed by Trivedi et al. (2021). That is, after pathogens trigger a significant increase in ROS, introduced antagonistic bacteria can restore cellular redox balance by secreting antioxidant molecules (such as glutathione and indole-3-acetic acid). However, the recovery of SOD activity by Bv+Rs treatment was limited (still lower than that in Bv treatment). Furthermore, the soluble sugar content in Bv+Rs treatment reached 6.03 mg g^-^¹, which was not significantly different from that in Bv treatment. This indicates that antagonistic bacteria can alleviate the pathogen’s plunder of carbon sources by promoting glycolysis and photosynthetic carbon assimilation (Stone et al., 2021). Bv treatment maintained the highest soluble sugar levels in both rounds of sampling and was significantly positively correlated with POD activity (r = 0.91, *p*< 0.01). This result supports the “sugar signaling-ROS interaction” hypothesis proposed by Keunen et al (Keunen et al., 2013). Soluble sugars not only act as osmotic regulators but also induce antioxidant enzyme gene expression by activating NADPH oxidase RBOH to produce trace amounts of H_2_O_2_. In contrast, Rs treatment inhibited the accumulation of soluble sugars, leading to weakened ROS scavenging capacity and ultimately increased MDA.

### Synergistic enhancement of soil nitrogen, phosphorus, potassium, and sulfur availability by *B. velezensis* M03

Exogenous microorganisms need to go through a colonization and adaptation period when entering the soil. During this period, they will face pressures such as protozoan predation and competition from indigenous microorganisms, and do not have the buffering capacity to adapt to the soil environment. Secondly, only when the population size reaches a threshold and functional genes are fully expressed can the secreted organic acids and extracellular enzymes break through the soil buffering system, leading to a rapid increase in available nutrients (Liu et al., 2026). This is consistent with the temporal pattern reported by Wang et al. that *Bacillus velezensis* starts 1~3 days after inoculation and changes properties 15~30 days after inoculation (Wang et al., 2024). At the same time, changes in the soil environment are not only affected by antagonistic bacteria; the change in microbial community functions after the introduction of antagonistic bacteria can also cause changes in soil chemical properties (Wang et al., 2025), that is, the comprehensive metabolic activity of the newly formed microbial community accelerates organic matter decomposition and nutrient cycling.

#### *B. velezensis* M03-driven soil nitrogen speciation

The Rs treatment significantly increased soil ammonium nitrogen (NH_4_^+^-N), while the Bv treatment significantly increased nitrate nitrogen (NO_3_^-^-N). *B. velezensis* M03 may have promoted rapid microbial assimilation of NH_4_^+^, thus reducing the risk of leaching, and subsequently released NO_3_^-^ for plant uptake through nitrification (Jia and Conrad, 2010). Notably, the Bv+Rs treatment significantly decreased NH_4_^+^ and increased NO_3_^-^ in both measurements, suggesting that strain M03 may carry more active ammonia oxidation-related genes (Leininger et al., 2006). This result is consistent with the phenomenon reported by Verhamme (Verhamme et al., 2011) of “rapid decrease of NH_4_^+^ and rapid accumulation of NO_3_^-^ after inoculation with ammonia-oxidizing bacteria”.

#### Microbial dissolution and chelation of phosphorus and potassium availability and their role in the sulfur cycle

Rs treatment resulted in the lowest levels of available phosphorus (AP) and available potassium (AK), while Bv and Bv+Rs treatments significantly increased AP and AK. This aligns with the “microbial phosphorus-potassium solubilization” mechanism proposed by Richardson & Simpson, whereby MO3 may promote the dissolution of insoluble phosphorus by chelating Ca²^+^, Al³^+^, and Fe³^+^ through the secretion of organic acids such as gluconic acid and oxalic acid (Richardson and Simpson, 2011). In addition, the weathering effect of Bv treatment on potassium minerals is consistent with the phenomenon reported by Sheng that “silicate bacteria form microdomain acidification via extracellular polysaccharides”, which provides strong evidence for explaining that Bv treatment increased the available potassium (AK) content by 75% (Sheng and He, 2006).Available sulfur (AS) reached 238.5 mg kg^-^¹ under *B. velezensis* (Bv) treatment, 4.8 times that of Rs treatment. This aligns with the pathway reported by Kertesz & Mirleau, where sulfur-oxidizing bacteria oxidize sulfur to SO_4_²^-^ via the sox gene cluster (Kertesz and Pascal, 2004). The Bv+Rs treatment increased the previously suppressed AS to 196.8 mg kg^-^¹. This further suggests that M03 may possess sulfur-oxidizing genes, which can work synergistically with other microorganisms to alleviate sulfur limitation and promote sulfur nutrition in plants.

#### *B. velezensis* M03 promotes a positive feedback loop in soil microbial-nutrient formation cycle

Phosphatase, urease, and leucine aminopeptidase activities were significantly higher under Bv treatment or Bv+Rs treatment than under Rs treatment. This indicates upregulation of functional gene expression in the microbial community, with phosphatase activity showing a significant positive correlation with available phosphorus and nitrate nitrogen (r > 0.82, *p*< 0.05). This supports the idea that microorganisms promote nutrient cycling through enzymatic hydrolysis (Burns et al., 2013). Urease activity showed a significant negative correlation with NH_4_^+^-N (r = -0.78, *p*< 0.05), further validating the rapid turnover of urea by microorganisms. The positive correlation between leucine aminopeptidase and available sulfur and total phosphorus (r > 0.75, *p*< 0.05) suggests that *B. velezensis* M03 may degrade organic nitrogen-sulfur complexes through secreted peptidase, simultaneously releasing sulfur and phosphorus. This is consistent with the phenomenon of “organic nitrogen mineralization accompanied by sulfur release” reported by Geisseler (Geisseler et al., 2011).

Two RDA results showed that the cumulative D explained by principal components 1 and 2 increased from 83.45% to 90.48%. This indicates that Bv and Bv+Rs treatments significantly altered the coupling relationship between soil chemistry and enzyme activity by regulating nitrogen, phosphorus, potassium, and sulfur cycles (Lauber et al., 2009). Notably, compared to the results of the first round of sampling (first round of sampling, 3 d), the correlation between phosphatase and urease activities and available phosphorus and nitrate nitrogen was enhanced (|r| > 0.85). Meanwhile, the correlation between peptidase and available sulfur and total phosphorus was enhanced (|r| > 0.80), indicating that the expression of microbial functional genes was further strengthened over time, forming a “positive feedback” cycle and promoting efficient nutrient utilization.

### *B. velezensis* M03 constructs a disease-resistant environmental barrier by optimizing the soil microecology

#### Response of soil microenvironment and functional communities

Rs treatment significantly promoted the abnormal proliferation of *Ralstonia*, the causal agent of bacterial wilt, with an increase of hundreds of times compared to the control group. This explosive growth of *Ralstonia* may be related to soil microenvironmental imbalance, providing a specific carbon source or altering the composition of root exudates (Wei et al., 2011). Notably, Bv+Rs treatment significantly inhibited *Ralstonia* proliferation while promoting the growth of beneficial microbial communities such as *Streptomyces* and *Trichoderma*. This result may stem from the competitive exclusion effect introduced by Bv and the antibiotic inhibition mechanism; Bv+Rs treatment effectively regulated community structure through microbial interactions.

The relative abundance of *Chloroflexota* decreased by 50.90% in the Rs treatment. This group is mostly photosynthetic autotrophic or anaerobic degradative bacteria. It is relatively sensitive to changes in soil redox potential and organic matter composition (Hug et al., 2013). *R. solanacearum* may have affected its living environment by altering pore structure or oxygen diffusion rate. Conversely, *Chloroflexota* remained stable in the CK control group, indicating that conventional management is more conducive to maintaining the ecological function of this group. The response of the fungal community was also significant. *Ascomycota* was highly enriched in the Bv+Rs treatment. Simultaneously, *Pseudallescheria*, which has the ability to decompose cellulose, and the antagonistic bacterium *Trichoderma* increased synergistically, suggesting that the Bv+Rs treatment may have promoted the development of saprophytic fungal functional groups by providing lignocellulosic substrates (Tedersoo et al., 2014).

Meanwhile, compared to the Rs treatment, where the relative abundance of *R. solanacearum* remained consistently high without significant fluctuations, the Bv+Rs treatment significantly reduced the relative abundance of *R. solanacearum*. Although the relative abundance of *B. velezensis* M03 decreased significantly over time, the relative abundance of *R. solanacearum* did not rebound as expected; instead, it showed a trend of continued suppression. Based on the above results, we propose the following speculations regarding the antagonistic mechanism of *B. velezensis* M03:First, *B. velezensis* M03 establishes initial inhibition against *R. solanacearum* through rapid competition for nutrients and ecological niches in the early stages of colonization. Second, *B. velezensis* M03 may exert sustained suppression on the pathogen by secreting antimicrobial substances (such as lipopeptide antibiotics) or inducing systemic resistance (ISR) (Keshmirshekan et al., 2024). These growth-independent mechanisms allow the antagonistic effect to be maintained even when the population of *B. velezensisima* M03 declines. This represents a significant ecological advantage compared to some strains that require high population densities to survive. Combined with the results of microbial community diversity, the bacterial community exhibited a more sensitive response to the disturbance caused by the antagonistic bacterium and *R. solanacearum*. The antagonistic bacterium could alleviate the negative impact of the pathogen on bacterial diversity in the early stage, whereas long-term coexistence with the pathogen conversely inhibited bacterial richness. Although the fungal community showed a weaker response, the combined treatment significantly reduced its diversity and richness at the later stage and drove its succession toward a unique community structure.

#### *B. velezensis* M03 mitigates the decay of deterministic processes in microbial community building and enhances its functional stability

For both bacteria and fungi, the linear association between species prevalence and mean relative abundance showed a slight decay at 10 d (bacteria: 0.814 → 0.801; fungi: 0.684 → 0.637). This suggests that repeated perturbations (pathogen invasion or antagonistic bacterial introduction) may weaken the deterministic process of community building, relatively enhancing the stochastic diffusion dynamics (Dini-Andreote et al., 2015). Abundance changes in the core taxa (persistent species) still have indicative significance for overall community stability. First, at the bacterial level, only the Bv+Rs treatment slightly increased the relative abundance of core species from 73.70% to 74.04%. Pathogen (Rs) decreased by 9.98%, indicating that *B. velezensis* M03 can partially buffer the negative selection pressure of *R. solanacearum* on the bacterial core (Wang et al., 2025). Second, the fungal core responded relatively weakly to the same treatment. Only the Bv+Rs treatment showed a recovery trend consistent with that of the bacteria. This further demonstrates the universal potential of *B. velezensis* M03 in maintaining the stability of microbial communities.

Generalized species dominated all samples, consistent with common characteristics of disturbed soils (Fierer, 2017). Notably, rare bacterial species only showed an increase in relative abundance in the Bv+Rs treatment (3.21% vs CK 1.99%). The highest value for rare fungi was observed in the CK treatment (12.29%), indicating that the response directions of rare bacterial and fungal taxa to the same intervention are not the same (Vandenkoornhuyse et al., 2015). Both Rs and Bv treatments resulted in a decrease in the relative abundance of rare bacteria, with similar reduction rates. This suggests that the introduction of exogenous strains, whether pathogenic or antagonistic, may “filter out” some low-abundance taxa through resource competition or antimicrobial effects, while combined treatments mitigate this effect through niche complementarity or antagonistic balance (Lennon and Jones, 2011).

Functional gene prediction results further revealed the functional characteristics of *B. velezensis* M03. Compared to the Rs treatment, the Bv+Rs treatment significantly reduced the functional abundance of plant pathogens without inducing a compensatory increase in new potential pathogen functions. This indicates that *B. velezensis* M03 can functionally weaken the pathogenic potential of *R. solanacearum* without introducing additional pathogen risks. Nitrogen cycle-related functions were enhanced only in the Bv+Rs treatment, suggesting a possible “co-stimulatory” effect between *R. solanacearum* and *B. velezensis* M03 (Truchon et al., 2022). This effect manifests in two ways: firstly, pathogen invasion leads to increased root permeability, providing substrates for ammonia-oxidizing and denitrifying microorganisms (Jaroentomeechai et al., 2025); secondly, *B. velezensis* enhances the activity of functional microorganisms by secreting auxins or improving oxygen permeability. Regarding fungal functional groups, Rs treatment enriched endophytic and epiphytic fungi, possibly because *R. solanacearum* infection caused root cell wall rupture, creating an entry point for the invasion and colonization of phyllosphere-rhizosphere fungi.

#### *Bacillus velezensis* M03 resists *Ralstonia solanacearum* invasion by restoring the microbial network structure

This study evaluated the antagonistic effect of *B. velezensis* M03 on peanut bacterial wilt. During the experimental period, neither the control (CK) nor the Bv treatment showed any symptoms of infection, indicating that *B. velezensis* M03 itself is non-pathogenic to peanut plants and does not induce the disease under normal environmental conditions. Notably, the Bv+Rs treatment exhibited a significant antagonistic effect against *R. solanacearum*. For example, 3 d after inoculation, the Bv+Rs treatment reduced the disease incidence from 50.00% in the Rs group to 16.67%, an inhibition rate of 66.67%. Simultaneously, the disease index also decreased from 16.67 to 4.17, an inhibition rate of 74.99%. This demonstrates that *B. velezensis* M03 significantly inhibited the occurrence and development of *R. solanacearum*.

Network analysis revealed that, compared to the control (CK) group, the Rs treatment significantly reduced the connectivity (number of edges and mean degree) of the bacterial network. This suggests that pathogen invasion may have disrupted the stable interspecies interactions within the original microbial community (Banerjee et al., 2018), leading to a simpler and more fragile network structure (Faust and Raes, 2012). The size and connectivity of the bacterial network treated with Bv+Rs almost recovered to the control level. This indicates that *B. velezensis* M03 effectively mitigated the destructive effects of *R. solanacearum*, helping the microbial community restore its complex interactions. This may be one of the key ecological mechanisms of its biocontrol efficacy (Hernandez et al., 2021). Furthermore, positive and negative correlations were roughly balanced in all bacterial networks, while fungal networks were generally dominated by extremely high positive correlations. This suggests that fungal communities in this system tend to co-economy in response to environmental pressures (Shen et al., 2023). In terms of modularity, the Bv treatment resulted in the highest degree of modularity in the bacterial network. This indicates that it can promote the formation of clearer and more stable functional modules in the community, which may enhance the community’s functional redundancy and resistance (Wei et al., 2015). This difference may be driven by the specific perturbation of the soil microenvironment by M03 and R. solanacearum. M03 has significantly higher abundance of functional genes related to nitrogen fixation, cellulose decomposition, and antibiotic synthesis. *R. solanacearum*, on the other hand, exhibits a typical “stress response” pattern in bacterial function (with more enrichment of genes related to nitrate reduction and metal resistance).

## Conclusion

*B. velezensis* M03 can directly and efficiently inhibit the occurrence and spread of *Ralstonia solanacearum*, reducing the incidence and disease index. In terms of peanut stress resistance, it significantly enhances the activity of SOD, POD, and CAT in peanut leaves, strengthening reactive oxygen species scavenging capacity, effectively mitigating membrane lipid peroxidation damage, and enhancing peanut stress resistance. At the soil microecological level, *B. velezensis* M03 drives the transformation and availability of key nutrients such as nitrogen, phosphorus, potassium, and sulfur. It optimizes the coupling relationship between soil chemistry and enzyme activity and promotes a stable microbial community structure composed of core taxa and complex networks, effectively resisting disturbances caused by pathogen invasion. *B. velezensis* M03 effectively inhibits the proliferation of *Ralstonia solanacearum*, promotes the growth of beneficial bacteria, maintains the abundance and network connectivity of core bacterial taxa, and enhances the modular structure and functional stability of the bacterial network. In summary, *B. velezensis* M03 can enhance peanut stress resistance and optimize soil microecology by synergistically enhancing plant antioxidant defense, optimizing soil nutrient cycling function, and reshaping the microbial interaction network. The data from this study provide a theoretical basis and practical support for developing integrated management strategies for soil diseases based on beneficial microorganisms.

## Data Availability

The datasets presented in this study can be found in online repositories. The names of the repository/repositories and accession number(s) can be found below: https://www.cncb.ac.cn/search?dbId=&q=PRJCA053568,PRJCA053568 https://www.cncb.ac.cn/search?dbId=&q=PRJCA058711,PRJCA058711.

## Supplementary material

Supplementary Table 1 Peanut plant disease severity assessment table.

Supplementary Table 2 The effect of *B. velezensis* M03 on the incidence of bacterial wilt in peanuts.

Supplementary Table 3 Physiological indicators of leaves in different treatment groups.

Supplementary Table 4 Effects of *B. velezensis* M03 on soil chemical indicators.

Supplementary Table 5 Effects of *B. velezensis* M03 treatment on soil enzyme activity.

Supplementary Table 6 Sequencing quality.

Supplementary Table 7 Effects of *B. velezensis* M03 treatment on microbial diversity.

Supplementary Table 8 Relative abundance at the bacterial phylum level(%).

Supplementary Table 9 Relative abundance at the bacterial genus level(%).

Supplementary Table 10 Relative abundance at the fungal phylum level(%).

Supplementary Table 11 Relative abundance at the fungal genus level(%).

Supplementary Table 12 Changes in the relative abundance of *Bacillus velezensis* M03 and *Ralstonia solanacearum*.

Supplementary Table 13 Bacterial community network topology index.

Supplementary Table 14 Fungal community network topology index.

Supplementary Figure 1 Principal component analysis of microbial communities.

## References

[B1] Bakr R. Abdelmoteleb A. Mendez-Trujillo V. Gonzalez-Mendoza D. Hewedy O. (2025). The potential of beneficial microbes for sustainable alternative approaches to control phytopathogenic diseases. Microbiol. Res. 16, 105. doi: 10.3390/microbiolres16050105. PMID: 41725453

[B2] Banerjee S. Schlaeppi K. van der Heijden M. G. A. (2018). Keystone taxa as drivers of microbiome structure and functioning. Nat. Rev. Microbiol. 16, 567–576. doi: 10.1038/s41579-018-0024-1. PMID: 29789680

[B3] Burns R. G. DeForest J. L. Marxsen J. Sinsabaugh R. L. Stromberger M. E. Wallenstein M. D. . (2013). Soil enzymes in a changing environment: current knowledge and future directions. Soil Biol. Biochem. 58, 216–234. doi: 10.1016/j.soilbio.2012.11.009. PMID: 41862359

[B4] Cao L. Chen J. Thia J. A. Schmidt T. L. Ffrench-Constant R. Zhang L. . (2025). Recurrent mutations drive the rapid evolution of pesticide resistance in the two-spotted spider mite tetranychus urticae. Elife 14, RP106288. doi: 10.7554/eLife.106288. PMID: 40788297 PMC12339004

[B5] Chen T. Yang W. Zhang H. Zhu B. Zeng R. Wang X. . (2020). Early detection of bacterial wilt in peanut plants through leaf-level hyperspectral and unmanned aerial vehicle data. Comput. Electron. Agric. 177, 105708. doi: 10.1016/j.compag.2020.105708. PMID: 41862359

[B6] Dini-Andreote F. Stegen J. C. van Elsas J. D. Salles J. F. (2015). Disentangling mechanisms that mediate the balance between stochastic and deterministic processes in microbial succession. Proc. Natl. Acad. Sci. U.S.A. 112, E1326–E1332. doi: 10.1073/pnas.1414261112. PMID: 25733885 PMC4371938

[B7] Du L. Huang X. Ding L. Wang Z. Tang D. Chen B. . (2023). TaERF87 and TaAKS1 synergistically regulate TaP5CS1 / TaP5CR1 ‐mediated proline biosynthesis to enhance drought tolerance in wheat. New Phytol. 237, 232–250. doi: 10.1111/nph.18549. PMID: 36264565

[B8] Edgar R. C. (2010). Search and clustering orders of magnitude faster than BLAST. Bioinformatics 26, 2460–2461. doi: 10.1093/bioinformatics/btq461. PMID: 20709691

[B9] Faust K. Raes J. (2012). Microbial interactions: from networks to models. Nat. Rev. Microbiol. 10, 538–550. doi: 10.1038/nrmicro2832. PMID: 22796884

[B10] Fierer N. (2017). Embracing the unknown: disentangling the complexities of the soil microbiome. Nat. Rev. Microbiol. 15, 579–590. doi: 10.1038/nrmicro.2017.87. PMID: 28824177

[B11] Geisseler D. Horwath W. R. Joergensen R. G. Ludwig B. (2011). Pathways of nitrogen utilization by soil microorganisms – a review. Soil Biol. Biochem. 42, 2058–2067. doi: 10.1016/j.soilbio.2010.08.021. PMID: 41862359

[B12] Glandorf D. C. M. Verheggen P. Jansen T. Jorritsma J. Smit E. Leeflang P. . (2001). Effect of genetically modified pseudomonas putida WCS358r on the fungal rhizosphere microflora of field-grown wheat. Appl. Environ. Microbiol. 67, 3371–3378. doi: 10.1128/AEM.67.8.3371-3378.2001. PMID: 11472906 PMC93030

[B13] Gu Y. B. Meng D. L. Yang S. Xiao N. W. Li Z. Y. Liu Z. H. . (2019). Invader-resident community similarity contribute to the invasion process and regulate biofertilizer effectiveness. J. Clean. Prod. 241, 118278. doi: 10.1016/j.jclepro.2019.118278. PMID: 41862359

[B14] Guo H. Yao J. Cai M. M. Qian Y. Guo Y. Richnow H. H. . (2012). Effects of petroleum contamination on soil microbial numbers, metabolic activity and urease activity. Chemosphere 87, 1273–1280. doi: 10.1016/j.chemosphere.2012.01.034. PMID: 22336736

[B15] Hernandez D. J. David A. S. Menges E. S. Searcy C. A. Afkhami M. E. (2021). Environmental stress destabilizes microbial networks. ISME J. 15, 1722–1734. doi: 10.1038/s41396-020-00882-x. PMID: 33452480 PMC8163744

[B16] Hou Q. Wang W. X. Yang Y. Hu J. Bian C. Jin L. . (2020). Rhizosphere microbial diversity and community dynamics during potato cultivation. Eur. J. Soil Biol. 98, 103176. doi: 10.1016/j.ejsobi.2020.103176. PMID: 41862359

[B17] Hu J. Wei Z. Friman V. Gu S. Wang X. Eisenhauer N. . (2016). Probiotic diversity enhances rhizosphere microbiome function and plant disease suppression. Mbio 7, e01716-e01790. doi: 10.1128/mBio.01790-16. PMID: 27965449 PMC5156302

[B18] Hu X. Shen S. Wu J. Liu J. Wang H. He J. . (2023). A natural allele of proteasome maturation factor improves rice resistance to multiple pathogens. Nat. Plants 9, 228–237. doi: 10.1038/s41477-022-01327-3. PMID: 36646829

[B19] Hug L. A. Castelle C. J. Wrighton K. C. Thomas B. C. Banfield J. F. (2013). Community genomic analyses constrain the distribution of metabolic traits across the chloroflexi phylum and indicate roles in sediment carbon cycling. Microbiome 1, 22. doi: 10.1186/2049-2618-1-22. PMID: 24450983 PMC3971608

[B20] Jaroentomeechai T. Veloz B. Soares C. O. Goerdeler F. Grosso A. S. Bull C. . (2025). Microbial binding module employs sophisticated clustered saccharide patches to selectively adhere to mucins. Nat. Commun. 16, 9058. doi: 10.1038/s41467-025-63756-w. PMID: 41083434 PMC12518865

[B21] Jia Z. Conrad R. (2010). Bacteria rather than archaea dominate microbial ammonia oxidation in an agricultural soil. Environ. Microbiol. 11, 1658–1671. doi: 10.1111/j.1462-2920.2009.01891.x. PMID: 19236445

[B22] Johansen A. Knudsen I. Binnerup S. Winding A. Johansen J. Jensen L. . (2005). Non-target effects of the microbial control agents DR54 and IK726 in soils cropped with barley followed by sugar beet: a greenhouse assessment. Soil Biol. Biochem. 37, 2225–2239. doi: 10.1016/j.soilbio.2005.04.004. PMID: 41862359

[B23] Jousset A. Schmid B. Scheu S. Eisenhauer N. (2011). Genotypic richness and dissimilarity opposingly affect ecosystem functioning. Ecol. Lett. 14, 537–545. doi: 10.1111/j.1461-0248.2011.01613.x. PMID: 21435139

[B24] Kertesz M. A. Pascal M. (2004). The role of soil microbes in plant sulphur nutrition. J. Exp. Bot. 55, 1939–1945. doi: 10.1093/jxb/erh176. PMID: 15181108

[B25] Keshmirshekan A. de Souza Mesquita L. M. Ventura S. P. M. (2024). Biocontrol manufacturing and agricultural applications of bacillus velezensis. Trends Biotechnol. 42, 986–1001. doi: 10.1016/j.tibtech.2024.02.003. PMID: 38448350

[B26] Keunen E. Peshev D. Vangronsveld J. Van den ende W. Cuypers A. (2013). Plant sugars are crucial players in the oxidative challenge during abiotic stress: extending the traditional concept. Plant Cell Environ. 36, 1242–1255. doi: 10.1111/pce.12061. PMID: 23305614

[B27] Klasek S. A. Brock M. T. Calder W. J. Morrison H. G. Weinig C. Maïgnien L. (2022). Spatiotemporal heterogeneity and intragenus variability in rhizobacterial associations with brassica rapa growth. Msystems 7, e00022-e00060. doi: 10.1128/msystems.00060-22. PMID: 35575562 PMC9239066

[B28] Lauber C. L. Hamady M. Knight R. Fierer N. (2009). Pyrosequencing-based assessment of soil ph as a predictor of soil bacterial community structure at the continental scale. Appl. Environ. Microbiol. 75, 5111–5120. doi: 10.1128/AEM.00335-09. PMID: 19502440 PMC2725504

[B29] Leininger S. Urich T. Schloter M. Schwark L. Qi J. Nicol G. W. . (2006). Archaea predominate among ammonia-oxidizing prokaryotes in soils. Nature 442, 806–809. doi: 10.1038/nature04983. PMID: 16915287

[B30] Lennon J. T. Jones S. E. (2011). Microbial seed banks: the ecological and evolutionary implications of dormancy. Nat. Rev. Microbiol. 9, 119–130. doi: 10.1038/nrmicro2504. PMID: 21233850

[B31] Li L. Q. Parts L. Madgwick P. King K. Flemming A. Woollard A. (2025). A proof-of-concept experimental-theoretical model to predict pesticide resistance evolution. Heredity. 1–12. doi: 10.1038/s41437-025-00781-x. PMID: 40702313 PMC7619028

[B32] Li T. Shi X. Wang J. Zhou Y. Wang T. Xu Y. . (2025). Turning antagonists into allies: bacterial-fungal interactions enhance the efficacy of controlling fusarium wilt disease. Sci. Adv. 11, eads5089. doi: 10.1126/sciadv.ads5089. PMID: 39937904 PMC11817942

[B33] Lian Q. Liu W. Ma D. Liang Z. Tang Z. Cao J. . (2023). Precisely orientating atomic array in one-dimension tellurium microneedles enhances intrinsic piezoelectricity for an efficient piezo-catalytic sterilization. ACS Nano 17, 8755–8766. doi: 10.1021/acsnano.3c02044. PMID: 37070712

[B34] Ling L. Han X. Li X. Zhang X. Wang H. Zhang L. . (2020). A streptomyces sp. NEAU-HV9: isolation, identification, and potential as a biocontrol agent against ralstonia solanacearum of tomato plants. Microorganisms 8, 351. doi: 10.3390/microorganisms8030351. PMID: 32121616 PMC7142955

[B35] Liu L. Ma L. Zhu M. Liu B. Liu X. Shi Y. (2023). Rhizosphere microbial community assembly and association networks strongly differ based on vegetation type at a local environment scale. Front. Microbiol. 14. doi: 10.3389/fmicb.2023.1129471. PMID: 36998396 PMC10043216

[B36] Liu Z. X. Gu H. D. Hu X. J. Yu Z. H. Li Y. S. Jin J. . (2026). Enhancing microbial carbon use efficiency in organic rice farming through improved soil nutrient availability and microbial resource acquisition strategies. Agr Ecosyst Environ. 397, 110097. doi: 10.1016/j.agee.2025.110097. PMID: 38423323

[B37] Ma B. Wang H. Dsouza M. Lou J. He Y. Dai Z. . (2016). Geographic patterns of co-occurrence network topological features for soil microbiota at continental scale in eastern China. ISME J. 10, 1891–1901. doi: 10.1038/ismej.2015.261. PMID: 26771927 PMC5029158

[B38] Magoč T. Salzberg S. L. (2011). FLASH: fast length adjustment of short reads to improve genome assemblies. Bioinformatics 27, 2957–2963. doi: 10.1093/bioinformatics/btr507. PMID: 21903629 PMC3198573

[B39] Qiong W. M G. G. M T. J. R C. J. (2007). Naive Bayesian classifier for rapid assignment of rRNA sequences into the new bacterial taxonomy. Appl Environ Microb. 73, 5261–5267. doi: 10.1128/AEM.00062-07. PMID: 17586664 PMC1950982

[B40] Raaijmakers J. M. Vlami M. de Souza J. T. (2002). Antibiotic production by bacterial biocontrol agents. Antonie van Leeuwenhoek 81, 537–547. doi: 10.1023/A:1020501420831. PMID: 12448749

[B41] Richardson A. E. Simpson R. J. (2011). Soil microorganisms mediating phosphorus availability update on microbial phosphorus. Plant Physiol. 156, 989–996. doi: 10.1104/pp.111.175448. PMID: 21606316 PMC3135950

[B42] Saiya-Cork K. R. Sinsabaugh R. L. D R Z. (2002). The effects of long term nitrogen deposition on extracellular enzyme activity in an acer saccharum forest soil. Soil Biol. Biochem. 34, 1309–1315. doi: 10.1016/S0038-0717(02)00074-3. PMID: 41810138

[B43] Shen Z. Yu B. Shao K. Gao G. Tang X. (2023). Warming reduces microeukaryotic diversity, network complexity and stability. Environ. Res. 238, 117235. doi: 10.1016/j.envres.2023.117235. PMID: 37775010

[B44] Sheng X. F. He L. Y. (2006). Solubilization of potassium-bearing minerals by a wild-type strain of bacillus edaphicus and its mutants and increased potassium uptake by wheat. Can. J. Microbiol. 52, 66–72. doi: 10.1139/w05-117. PMID: 16541160

[B45] Stone B. W. Li J. Koch B. J. Blazewicz S. J. Dijkstra P. Hayer M. . (2021). Nutrients cause consolidation of soil carbon flux to small proportion of bacterial community. Nat. Commun. 12, 3381. doi: 10.1038/s41467-021-23676-x. PMID: 34099669 PMC8184982

[B46] Sun X. Xu Z. Xie J. Hesselberg-Thomsen V. Tan T. Zheng D. . (2022). Bacillus velezensis stimulates resident rhizosphere pseudomonas stutzeri for plant health through metabolic interactions. ISME J. 16, 774–787. doi: 10.1038/s41396-021-01125-3. PMID: 34593997 PMC8483172

[B47] Tedersoo L. Bahram M. Plme S. Kljalg U. Abarenkov K. (2014). Fungal biogeography. Global diversity and geography of soil fungi. Science 346, 1256688. doi: 10.1126/science.1256688. PMID: 25430773

[B48] Trivedi P. Mattupalli C. Eversole K. Leach J. E. (2021). Enabling sustainable agriculture through understanding and enhancement of microbiomes. New Phytol. 230, 2129–2147. doi: 10.1111/nph.17319. PMID: 33657660

[B49] Truchon A. N. Hendrich C. G. Bigott A. F. Dalsing B. L. Allen C. (2022). NorA, HmpX, and NorB cooperate to reduce NO toxicity during denitrification and plant pathogenesis in ralstonia solanacearum. Microbiol. Spectr. 10, e0026422. doi: 10.1128/spectrum.00264-22. PMID: 35377234 PMC9045102

[B50] Vandenkoornhuyse P. Quaiser A. Duhamel M. Le Van A. Dufresne A. (2015). The importance of the microbiome of the plant holobiont. New Phytol. 206, 1196–1206. doi: 10.1111/nph.13312. PMID: 25655016

[B51] Verhamme D. T. Prosser J. I. Nicol G. W. (2011). Ammonia concentration determines differential growth of ammonia-oxidising archaea and bacteria in soil microcosms. ISME J. 5, 1067–1071. doi: 10.1038/ismej.2010.191. PMID: 21228892 PMC3131854

[B52] Wang Q. Garrity G. M. Tiedje J. M. Cole J. R. (2007). Naïve bayesian classifier for rapid assignment of rRNA sequences into the new bacterial taxonomy. Appl. Environ. Microbiol. 73, 5261–5267. doi: 10.1128/AEM.00062-07. PMID: 17586664 PMC1950982

[B53] Wang X. Qi F. Sun Z. Liu H. Wu Y. Wu X. . (2024). Transcriptome sequencing and expression analysis in peanut reveal the potential mechanism response to ralstonia solanacearum infection. BMC Plant Biol. 24, 207. doi: 10.1186/s12870-024-04877-0. PMID: 38515036 PMC10956345

[B54] Wang S. H. Su C. Yang S. Q. Wang S. Jiang X. T. He H. L. . (2025). Synergistic co-inoculation of bacillus velezensis and pseudomonas helmanticensis enhances corn straw degradation via microbial community restructuring and saprotroph dominance. Microorganisms 13, 2612. doi: 10.3390/microorganisms13112612. PMID: 41304296 PMC12654394

[B55] Wang W. Xia Y. Zhang P. Zhu M. Huang S. Sun X. . (2025). Narrow-spectrum resource-utilizing bacteria drive the stability of synthetic communities through enhancing metabolic interactions. Nat. Commun. 16, 6088. doi: 10.1038/s41467-025-61432-7. PMID: 40603291 PMC12222865

[B56] Wang Y. Ying G. Zhang Z. Tang Y. Zhang Y. Chen L. (2024). Bacillus velezensis promotes the proliferation of lactic acid bacteria and influences the fermentation quality of whole-plant corn silage. Front. Plant Sci. 15. doi: 10.3389/fpls.2024.1285582. PMID: 38425795 PMC10902168

[B57] Warton D. I. Wright S. T. Wang Y. (2012). Distance‐based multivariate analyses confound location and dispersion effects. Methods Ecol. Evol. 3, 89–101. doi: 10.1111/j.2041-210X.2011.00127.x. PMID: 41858021

[B58] Wei Z. Yang T. Friman V. Xu Y. Shen Q. Jousset A. (2015). Trophic network architecture of root-associated bacterial communities determines pathogen invasion and plant health. Nat. Commun. 6, 8413. doi: 10.1038/ncomms9413. PMID: 26400552 PMC4598729

[B59] Wei Z. Yang X. Yin S. Shen Q. Ran W. Xu Y. (2011). Efficacy of bacillus-fortified organic fertiliser in controlling bacterial wilt of tomato in the field. Appl. Soil Ecol. 48, 152–159. doi: 10.1016/j.apsoil.2011.03.013. PMID: 41862359

[B60] Xu J. Xu J. Shi T. Zhang Y. Chen F. Yang C. . (2023). Probiotic‐inspired nanomedicine restores intestinal homeostasis in colitis by regulating redox balance, immune responses, and the gut microbiome. Adv. Mater. 35, 2207890. doi: 10.1002/adma.202207890. PMID: 36341495

[B61] Yilma E. Tadesse F. Alemu T. (2025). Biocontrol potential of bacillus and pseudomonas species against fusarium oxysporum, a causative agent of chili pepper wilt disease in Ethiopia. Discover Agric. 3, 261. doi: 10.1007/s44279-025-00445-8. PMID: 41859209

[B62] Zhang J. Zhang B. Liu Y. Guo Y. Shi P. Wei G. (2018). Distinct large-scale biogeographic patterns of fungal communities in bulk soil and soybean rhizosphere in China. Sci. Total Environ. 644, 791–800. doi: 10.1016/j.scitotenv.2018.07.016. PMID: 29990927

[B63] Zhao K. Li Y. Wang J. Tu Y. Cao Z. Ma X. . (2025). Genome-wide characterization of AhBAG genes in peanut reveals their role in bacterial wilt resistance and hormone response. BMC Plant Biol. 25, 513. doi: 10.1186/s12870-025-06552-4. PMID: 40269706 PMC12016183

[B64] Zhou X. Zhang J. Shi J. Khashi U Rahman M. Liu H. Wei Z. . (2024). Volatile-mediated interspecific plant interaction promotes root colonization by beneficial bacteria via induced shifts in root exudation. Microbiome 12, 207. doi: 10.1186/s40168-024-01914-w. PMID: 39428455 PMC11492557

