# Supplementary material for: Bacillus velezensis M03 enhances peanut stress resistance and optimizes soil microecology, thereby increasing its resistance to Ralstonia solanacearum
Source: Front Plant Sci. 2026 Apr 10;17:1788450. doi: 10.3389/fpls.2026.1788450 (PMC13106345; doi:10.3389/fpls.2026.1788450)
Supplement: Supplementary Table 1 — Peanut plant disease severity assessment table. [file DataSheet1.doc]

Table S1. Peanut Plant Disease Severity Assessment Table

| Disease severity | Evaluation criteria |
| --- | --- |
| Level 0 | The entire plant is disease-free, the leaves are normal, and there are no signs of wilting or yellowing. |
| Level 1 | The percentage of diseased leaves was ≤25%, with only a few bottom leaves showing slight wilting. |
| Level 2 | 5% < diseased leaves ≤ 50%, lower and middle leaves wilted. |
| Level 3 | 50% < diseased leaves ≤ 75%, most leaves wilted. |
| Level 4 | When more than 75% of the leaves are diseased, the entire plant's leaves wilt or die severely. |

Table S2. The effect of *B. velezensis* M03 on the incidence of bacterial wilt in peanuts

| Group | 3 d | | | | 10 d | | | |
| --- | --- | --- | --- | --- | --- | --- | --- | --- |
|  | Incidence rate | Disease index | Incidence suppression rate /% | Disease index inhibition rate /% | Incidence rate | Disease index | Incidence suppression rate/% | Disease index inhibition rate/% |
| CK | 0.00% | 0.00 | - | - | 0.00% | 0.00 | - | - |
| BV | 0.00% | 0.00 | - | - | 0.00% | 0.00 | - | - |
| Rs | 50.00% | 16.67 | - | - | 100.00% | 54.17 | - | - |
| Bv+Rs | 16.67% | 4.17 | 66.67% | 74.99% | 50.00% | 16.67 | 50.00% | 69.23% |

Table S3. Physiological indicators of leaves in different treatment groups

| sample | SOD/(U/mL) | POD/(U/mL) | CAT/(U/mL) | MDA/(nmol/mL) | Soluble sugar/(mg/g) |
| --- | --- | --- | --- | --- | --- |
| CK_1 | 158.13±14.50a | 2331.67±92.2d | 110.01±25.02c | 11.65±1.19b | 1.50±0.13c |
| Bv_1 | 554.04±89.51a | 5741.00±56.43a | 147.78±0.59a | 2.34±0.90c | 3.97±0.12a |
| Rs_1 | 121.56±3.93a | 1820.67±35.85e | 106.04±19.66c | 34.05±1.88a | 1.40±0.10c |
| Bv+Rs_1 | 452.29±12.39a | 5025.33±31.66b | 139.1±10.72ab | 16.55±6.16b | 2.86±1.14b |
| CK_2 | 14.96±0.86c | 6108.67±172.08b | 97.54±0.28d | 14.87±0.21e | 4.84±0.04c |
| Bv_2 | 141.56±17.45a | 12658.33±4186.94a | 139.80±0.12a | 11.10±0.14d | 6.25±0.06a |
| Rs_2 | 14.41±3.09c | 5357.33±2857.73b | 37.16±0.31e | 81.12±0.17a | 3.21±0.04d |
| Bv+Rs_2 | 29.67±0.99b | 8134±129.64ab | 130.98±0.44b | 18.56±0.18c | 6.03±0.02b |

Note: Different lowercase letters indicate significant differences among groups of the same index at the same treatment day (*p* < 0.05).

Table S4. Effects of *B. velezensis* M03 on soil chemical indicators

|  | Group | NH_4_^+^-N  /(μg/mL) | NO_3_^-^-N  /(μg/mL) | AP  /(μmol/mL) | TP  /(μg/g) | AS  /(mg/kg) | AK  /(mg/kg) |
| --- | --- | --- | --- | --- | --- | --- | --- |
| 3 d (First round of sampling) | CK_1 | 13.01±0.52c | 3.36±0.30c | 3.82±0.34a | 204.41±8.72b | 102.05±1.91c | 0.49±0.37a |
|  | Bv_1 | 13.25±1.10bc | 6.00±0.34a | 5.00±1.45a | 296.12±17.15a | 238.50±13.05a | 0.72±0.32a |
|  | Rs_1 | 15.99±1.83a | 1.70±0.29d | 2.14±0.39a | 176.97±16.97c | 49.50±0.89e | 0.44±0.28a |
|  | Bv+Rs_1 | 15.28±1.01ab | 5.41±0.43b | 5.16±4.87a | 282.59±13.17a | 200.50±7.58b | 0.62±0.31a |
| 10 d (Second round of sampling) | CK_2 | 9.86±0.18c | 2.87±0.03d | 3.33±0.19a | 189.46±13.04c | 100.40±1.25c | 0.04±0.00d |
|  | Bv_2 | 10.43±0.95c | 5.44±0.02a | 4.73±1.29a | 275.92±11.78a | 236.03±14.47a | 0.07±0.00a |
|  | Rs_2 | 13.27±0.10a | 1.21±0.02e | 1.72±0.14a | 155.39±5.81d | 46.83±0.58e | 0.04±0.00d |
|  | Bv+Rs_2 | 12.45±0.27b | 4.95±0.11b | 4.65±4.74a | 247.50±8.54b | 196.83±6.86b | 0.06±0.00b |

Note: Different lowercase letters indicate significant differences among groups of the same index at the same treatment day (*p* < 0.05).

Table S5. Effects of *B. velezensis* M03 treatment on soil enzyme activity

| 3 d (First round of sampling) | Group | S-ACP/(U/g) | S-UE/(U/g) | S-LAP/(U/g) |
| --- | --- | --- | --- | --- |
|  | CK_1 | 252921.40±748.23c | 5301.95±22.31b | 2.67±0.01b |
|  | Bv_1 | 282400.25±1311.38a | 7167.19±27.21a | 8.96±0.34a |
|  | Rs_1 | 250898.19±1277.48d | 1823.20±8.21e | 0.09±0.07e |
|  | Bv+Rs_1 | 253200.99±843.42c | 4483.44±17.46c | 1.00±0.02c |
| 10 d (Second round of sampling) | CK_2 | 194111.10±1846.71c | 2479.92±118.87d | 0.61±0.01c |
|  | Bv_2 | 245265.74±1210.00a | 5952.50±45.64a | 1.49±0.21a |
|  | Rs_2 | 193699.34±1462.06c | 1845.00±3.55e | 0.06±0.04d |
|  | Bv+Rs_2 | 243933.88±900.91a | 4310.70±13.20b | 1.10±0.09b |

Note: Different lowercase letters indicate significant differences among groups of the same index at the same treatment day (*p* < 0.05).

Table S6. Sequencing quality

| Group | | Bacteria | Fungus |
| --- | --- | --- | --- |
| 3 d (First round of sampling) | CK_1 | 411 | 112 |
|  | Bv_1 | 400 | 100 |
|  | Rs_1 | 376 | 102 |
|  | Bv+Rs_1 | 430 | 140 |
| 10 d (Second round of sampling) | CK_2 | 411 | 106 |
|  | Bv_2 | 453 | 153 |
|  | Rs_2 | 446 | 140 |
|  | Bv+Rs_2 | 375 | 86 |

Note: The data in the table are statistics of the number of observable bacterial and fungal genera in the corresponding samples.

Table S7. Effects of *B. velezensis* M03 treatment on microbial diversity

| Group | | Bacteria | | Fungi | |
| --- | --- | --- | --- | --- | --- |
|  |  | Shannon | Chao1 | Shannon | Chao1 |
| 3 d (First round of sampling) | CK_1 | 7.93±0.27ab | 2592.68±232.94ab | 3.88±0.17a | 338.46±32.61b |
|  | Bv_1 | 7.93±0.16ab | 2705.56±162.16ab | 3.46±0.21b | 322.88±36.96bc |
|  | Rs_1 | 7.36±0.17c | 2384.24±143.26bc | 3.63±0.13ab | 337.56±11.28bc |
|  | Bv+Rs_1 | 7.9±0.18ab | 2572.17±141.78ab | 3.99±0.44a | 325.11±27.55a |
| 10 d (Second round of sampling) | CK_2 | 7.75±0.81ab | 2509.42±500.5bc | 3.35±1.02b | 314.98±27.2c |
|  | Bv_2 | 8.16±0.32a | 2892.14±304.8a | 3.86±0.45a | 393.33±57.57b |
|  | Rs_2 | 7.63±0.33bc | 2914.71±86.37a | 3.96±0.38a | 399.47±24.75a |
|  | Bv+Rs_2 | 7.44±0.46bc | 2282.04±372.74c | 3.11±0.85b | 280.18±73.14c |

Note: Different lowercase letters indicate significant differences among groups of the same index at the same treatment day (*p* < 0.05).

Table S8. Relative abundance at the bacterial phylum level（%）

| group | CK_1 | Bv_1 | Rs_1 | Bv+Rs_1 | CK_2 | Bv_2 | Rs_2 | Bv+Rs_2 |
| --- | --- | --- | --- | --- | --- | --- | --- | --- |
| Pseudomonadota | 27.31 | 41.27 | 44.97 | **51.98** | 29.62 | 34.39 | **54.14** | 36.56 |
| Actinomycetota | 21.62 | 20.47 | 21.52 | 12.1 | 24.37 | 20.69 | 20.5 | 22.03 |
| Chloroflexota | 18.72 | 9.13 | **13.3** | 7.06 | 17.21 | 14.3 | **6.53** | 14.63 |
| Bacteroidota | 8.87 | 6.54 | 4.73 | 9.05 | 7.73 | 6.46 | 3.92 | 7.8 |
| Bacillota | 3.2 | 4.73 | 1.85 | 3.91 | 3.6 | 3.78 | 2.52 | 2.76 |
| Acidobacteriota | 5.6 | 3.33 | 2.84 | 3.05 | 4.05 | 4.18 | 2.6 | 2.97 |
| Bdellovibrionota | 2.24 | 5.35 | 3.68 | 5.57 | 1.56 | 1.98 | 2.1 | 2.07 |
| Patescibacteria | 2.99 | 2.52 | 2.13 | 1.78 | 3.16 | 2.66 | 2.1 | 3.67 |
| Verrucomicrobiota | 3.14 | 2.24 | 1.67 | 1.81 | 2.53 | 4.45 | 1.08 | 2.38 |
| Myxococcota | 1.88 | 1.48 | 0.78 | 0.89 | 2.18 | 3.34 | 1.12 | 1.71 |
| others | 4.43 | 2.93 | 2.54 | 2.8 | 3.99 | 3.78 | 3.38 | 3.42 |

Note: The data in the table are the relative abundances of the top 10 bacterial phyla in CK, Bv, Rs, and Bv+RS treatment groups at 3 d and 10 d.

Table S9. Relative abundance at the bacterial genus level（%）

| group | CK_1 | Bv_1 | Rs_1 | Bv+Rs_1 | CK_2 | Bv_2 | Rs_2 | Bv+Rs_2 |
| --- | --- | --- | --- | --- | --- | --- | --- | --- |
| Streptomyces | 0.104 | 0.084 | 0.082 | 0.035 | 0.129 | 0.077 | 0.046 | 0.130 |
| Ralstonia | 0.001 | 0.001 | 0.159 | 0.072 | 0.000 | 0.013 | **0.185** | 0.077 |
| Pseudomonas | 0.003 | 0.091 | 0.070 | 0.103 | 0.008 | 0.027 | 0.069 | 0.010 |
| Sphingomonas | 0.026 | 0.042 | 0.025 | 0.043 | 0.027 | 0.056 | 0.033 | 0.031 |
| Actinoallomurus | 0.049 | 0.041 | 0.040 | 0.015 | 0.045 | 0.034 | 0.039 | 0.034 |
| Devosia | 0.020 | 0.023 | 0.029 | 0.016 | 0.029 | 0.034 | 0.037 | 0.037 |
| Dictyobacter | 0.033 | 0.024 | 0.026 | 0.015 | 0.066 | 0.030 | 0.010 | 0.040 |
| Ensifer | 0.013 | 0.020 | 0.018 | 0.029 | 0.013 | 0.018 | 0.021 | 0.013 |
| Massilia | 0.005 | 0.014 | 0.046 | 0.035 | 0.010 | 0.008 | 0.026 | 0.003 |
| Bdellovibrio | 0.001 | 0.050 | 0.013 | 0.056 | 0.000 | 0.011 | 0.009 | 0.005 |
| others | 0.745 | 0.610 | 0.491 | 0.581 | 0.671 | 0.693 | 0.526 | 0.620 |

Note: The data in the table are the relative abundances of the top 10 bacterial genera in CK, Bv, Rs, and Bv+RS treatment groups at 3 d and 10 d.

Table S10. Relative abundance at the fungal phylum level（%）

| group | CK_1 | Bv_1 | Rs_1 | Bv+Rs_1 | CK_2 | Bv_2 | Rs_2 | Bv+Rs_2 |
| --- | --- | --- | --- | --- | --- | --- | --- | --- |
| Ascomycota | 46.71 | 54.68 | 54.80 | 62.81 | 71.44 | 45.89 | 43.72 | 74.75 |
| Basidiomycota | 49.41 | 43.63 | 43.27 | 34.36 | 26.47 | 52.61 | 50.11 | 23.13 |
| Chytridiomycota | 2.08 | 0.72 | 0.57 | 2.02 | 1.02 | 0.76 | 3.96 | 0.35 |
| Fungi | 1.75 | 0.95 | 1.33 | 0.68 | 0.84 | 0.67 | 2.09 | 1.68 |
| Mortierellomycota | 0.03 | 0.00 | 0.00 | 0.08 | 0.21 | 0.02 | 0.07 | 0.08 |
| Mucoromycota | 0.01 | 0.01 | 0.03 | 0.05 | 0.01 | 0.02 | 0.02 | 0.03 |
| Glomeromycota | 0.00 | 0.00 | 0.00 | 0.00 | 0.01 | 0.01 | 0.00 | 0.00 |
| Calcarisporiellomycota | 0.00 | 0.00 | 0.00 | 0.00 | 0.00 | 0.00 | 0.01 | 0.00 |
| Olpidiomycota | 0.00 | 0.00 | 0.00 | 0.00 | 0.00 | 0.00 | 0.03 | 0.00 |
| Aphelidiomycota | 0.00 | 0.00 | 0.00 | 0.00 | 0.01 | 0.00 | 0.00 | 0.00 |
| others | 0.00 | 0.00 | 0.00 | 0.00 | 0.00 | 0.03 | 0.00 | 0.00 |

Note: The data in the table are the relative abundances of the top 10 fungal phyla in CK, Bv, Rs, and Bv+RS treatment groups at 3 d and 10 d.

Table S11. Relative abundance at the fungal genus level（%）

| group | CK_1 | Bv_1 | Rs_1 | Bv+Rs_1 | CK_1 | Bv_2 | Rs_2 | Bv+Rs_2 |
| --- | --- | --- | --- | --- | --- | --- | --- | --- |
| Clitopilus | 40.80 | 34.97 | 36.35 | **29.54** | 15.91 | 42.33 | 31.06 | 15.27 |
| Pseudallescheria | 19.41 | 30.13 | 24.81 | 20.87 | 52.55 | 29.06 | 19.84 | **45.14** |
| Xylariales_gen_Incertae_sedis | 8.80 | 10.04 | 8.52 | 6.15 | 11.23 | 6.83 | 5.66 | 5.72 |
| Coniochaeta | 3.88 | 4.15 | 7.13 | 2.75 | 3.88 | 2.71 | 3.93 | 2.33 |
| Talaromyces | 0.59 | 0.12 | 0.12 | 5.96 | 0.15 | 0.10 | 0.18 | 0.07 |
| Myriococcum | 6.83 | 6.67 | 5.24 | 3.99 | 3.55 | 7.74 | 4.46 | 5.87 |
| Trichoderma | 2.67 | 3.31 | 3.59 | 3.79 | 2.54 | 2.19 | 2.75 | **18.53** |
| Phialemonium | 3.21 | 4.03 | 5.46 | 3.58 | 1.30 | 1.09 | 1.28 | 0.81 |
| Penicillium | 3.35 | 0.68 | 0.46 | 7.75 | 0.37 | 0.46 | 4.56 | 0.85 |
| Neocosmospora | 0.02 | 0.00 | 0.00 | 3.60 | 0.02 | 0.05 | 0.05 | 0.03 |
| others | 10.43 | 5.90 | 8.33 | 12.02 | 8.50 | 7.45 | **26.23** | 5.38 |

Note: The data in the table are the relative abundances of the top 10 fungal genera in CK, Bv, Rs, and Bv+RS treatment groups at 3 d and 10 d.

Table S12. Changes in the relative abundance of *Bacillus velezensis* M03 and *Ralstonia solanacearum*

| Group | *Bacillus velezensis*（M03） | *Ralstonia solanacearum* |
| --- | --- | --- |
| CK_1 | 0.00%±0.00b | 0.00%±0.00d |
| Bv_1 | 13.00%±0.08a | 0.00%±0.00d |
| Rs_1 | 1.00%±0.01b | 10.00%±0.01a |
| Bv+Rs_1 | 9.00%±0.02a | 5.00%±0.01b |
| CK_2 | 0.00%±0.00b | 0.00%±0.00d |
| Bv_2 | 9.00%±0.06a | 1.00%±0.01cd |
| Rs_2 | 0.00%±0.00b | 13.00%±0.08a |
| Bv+Rs_2 | 2.00%±0.02b | 4.00%±0.03bc |

Note: Different lowercase letters indicate significant differences in the relative abundance of the antagonistic bacterium *Bacillus velezensis* or the pathogenic bacterium *Ralstonia solanacearum* among CK, Bv, Rs, and Bv+RS treatments (*p* < 0.05).

Table S13. Bacterial community network topology index

| value | CK | Bv | Rs | Bv+Rs |
| --- | --- | --- | --- | --- |
| num edges | 7412 | 6592 | 6443.5 | 7195.5 |
| Pos/total edges(%) | 49.89% | 50.70% | 51.56% | 54.50% |
| average degree | 74.49 | 66.08 | 64.76 | 72.65 |
| relative modularity | 7.91 | 9.15 | 8.81 | 9.20 |

Note: The data in the table are the topological indices of the bacterial community network under CK, Bv, Rs, and Bv+RS treatments.

Table S14. Fungal community network topology index

| value | CK | Bv | Rs | Bv+Rs |
| --- | --- | --- | --- | --- |
| num edges | 5174 | 4616 | 5028 | 5241 |
| Pos/total edges (%) | 64.41 | 58.19 | 57.83 | 73.33 |
| average degree | 51.75 | 46.16 | 50.28 | 52.42 |
| relative modularity | 7.56 | 6.90 | 7.66 | 7.17 |

Note: The data in the table are the topological indices of the fungal community network under CK, Bv, Rs, and Bv+RS treatments.


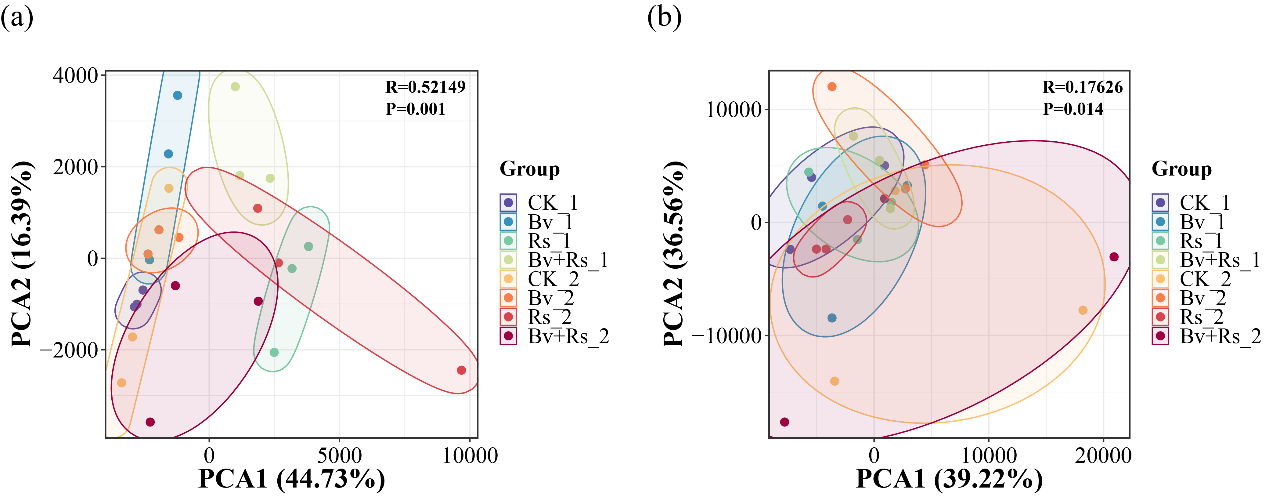


Note: (a) represents the bacterial community; (b) represents the fungal community.

Figure S1. Principal component analysis of microbial communities
